# Stepwise emergence of the neuronal gene expression program in early animal evolution

**DOI:** 10.1016/j.cell.2023.08.027

**Published:** 2023-10-12

**Authors:** Sebastián R. Najle, Xavier Grau-Bové, Anamaria Elek, Cristina Navarrete, Damiano Cianferoni, Cristina Chiva, Didac Cañas-Armenteros, Arrate Mallabiabarrena, Kai Kamm, Eduard Sabidó, Harald Gruber-Vodicka, Bernd Schierwater, Luis Serrano, Arnau Sebé-Pedrós

**Affiliations:** 1 Centre for Genomic Regulation (CRG), Barcelona Institute of Science and Technology (BIST), Barcelona, Spain; 2 Universitat Pompeu Fabra (UPF), Barcelona, Spain; 3 ICREA, Barcelona, Spain; 4 Institute of Animal Ecology, University of Veterinary Medicine Hannover, Foundation, Hannover, Germany; 5 Max Planck Institute for Marine Microbiology, Bremen, Germany; 6 Zoological Institute, Christian Albrechts University, Kiel, Germany; 7 American Museum of Natural History, Richard Gilder Graduate School, NY, USA

**Keywords:** evolution, developmental biology, biodiversity, neuroscience, cell differentiation, chromatin biology, phylogenetics, single-cell transcriptomics, comparative genomics, Notch signaling

## Abstract

The assembly of the neuronal and other major cell type programs occurred early in animal evolution. We can reconstruct this process by studying non-bilaterians like placozoans. These small disc-shaped animals not only have nine morphologically described cell types and no neurons but also show coordinated behaviors triggered by peptide-secreting cells. We investigated possible neuronal affinities of these peptidergic cells using phylogenetics, chromatin profiling, and comparative single-cell genomics in four placozoans. We found conserved cell type expression programs across placozoans, including populations of transdifferentiating and cycling cells, suggestive of active cell type homeostasis. We also uncovered fourteen peptidergic cell types expressing neuronal-associated components like the pre-synaptic scaffold that derive from progenitor cells with neurogenesis signatures. In contrast, earlier-branching animals like sponges and ctenophores lacked this conserved expression. Our findings indicate that key neuronal developmental and effector gene modules evolved before the advent of cnidarian/bilaterian neurons in the context of paracrine cell signaling.

## Highlights

• Comparative single-cell genomics reveals cell type diversity in the phylum Placozoa
• Fourteen placozoan peptidergic cell types expressing neuronal genes
• Post-translationally modified neuropeptides define elaborate cell signaling network
• Peptidergic progenitors with neurogenesis-like differentiation from epithelial cells

## Graphical abstract

**Figure:**
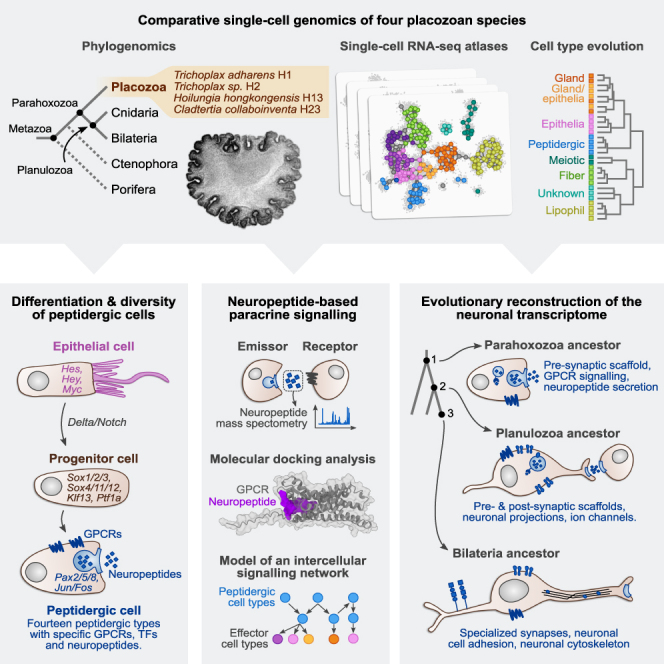

## Teaser

A comparative study of cell diversity in placozoans indicates that key neuronal modules evolved before the advent of cnidarian/bilaterian neurons in the context of paracrine cell signaling.

## Introduction

The division of functions betwen cell types is a hallmark of animal multicellularity.^1^^,^^2^^,^^3^ Specialized cell states result from the differential co-regulation of functional gene modules, such as actomyosin contractility, ciliary, or pre-synaptic scaffold components.^3^^,^^4^ Through comparative genomics, we have a detailed understanding of the evolutionary histories of the constituents of these modules, with many key animal genes predating multicellularity.^2^^,^^5^ In contrast, we lack a detailed understanding of when these genes assembled into co-regulated modules, how they are deployed in different cell types, and what their evolutionary dynamics are. The comparative study of cell diversity and genome regulation in early-branching, non-bilaterian animals (sponges, cnidarians, ctenophores, and placozoans) offers the opportunity to address these fundamental questions and reconstruct the evolutionary emergence of major cell type programs.

Placozoans are millimeter-sized, flat animals that employ ciliary beating and mucus secretion to glide over surfaces.^6^^,^^7^^,^^8^^,^^9^ These marine animals feed on algae and other microbial eukaryotes by extracellular lysis of prey, phagocytosis, and intracellular digestion.^10^ Placozoans emerged 750–800 mya^11^^,^^12^ and their known diversity is small, with four described species and a dozen additional known mitochondrial haplotypes, some of which likely represent additional species.^7^^,^^12^^,^^13^ Placozoan genomes are compact (87–105 Mb), but they encode a conserved repertoire of genes involved in signaling and regulatory functions, shared with cnidarians and bilaterians.^14^ Unlike in other animals, gene regulation in placozoans is controlled by proximal promoter elements, with no distal enhancers.^15^

The placozoan body plan consists of two cell layers and six to nine major somatic cell types. The lower epithelium features gland cells that produce mucus and contain secretory granules^16^ and lipophil cells involved in algal digestion that contain large lipophilic granules.^10^ Gland and lipophil cells are embedded between narrow ciliated cells responsible for animal gliding and food absorption.^10^^,^^16^ The upper epithelium is also ciliated, but cells are flat and large,^17^ and some contain auto-fluorescent shiny spheres involved in predator defense.^18^ Connecting between these two epithelial layers are amoeboid-shaped fiber cells with long filopodia that are responsible for contractility and, possibly, phagocytosis.^19^ The collective behavior of placozoan cells is controlled by paracrine signaling,^20^ via small neuropeptides (NPs) secreted by low-abundance peptidergic cells that lack cellular projections and synapses. Examples of such behaviors are contractility and detachment induced by SIFGamide and PWN peptides,^21^ as well as ciliary beating in the lower epithelial layer controlled by ELPE and FFNPamide.^21^^,^^22^ Because of the presence of these peptidergic cells and NP-driven behaviors, the study of placozoans can provide insights into early steps in neuronal evolution. Thus, we decided to dissect the molecular diversity of placozoans cell types and compare them with other species.

To understand the cellular diversity in placozoans, here we used single-cell transcriptomics to characterize cell type gene expression and cell differentiation dynamics across four species. We combined these expression maps with genome-wide profiling of *cis*-regulatory elements (REs) to decode regulatory programs in placozoans. Finally, we conducted cross-species comparative analyses to reconstruct the evolution of placozoan gene modules and, ultimately, the emergence of the neuronal gene expression program.

## Results

### Placozoa phylogenomics

**Figure 1:**
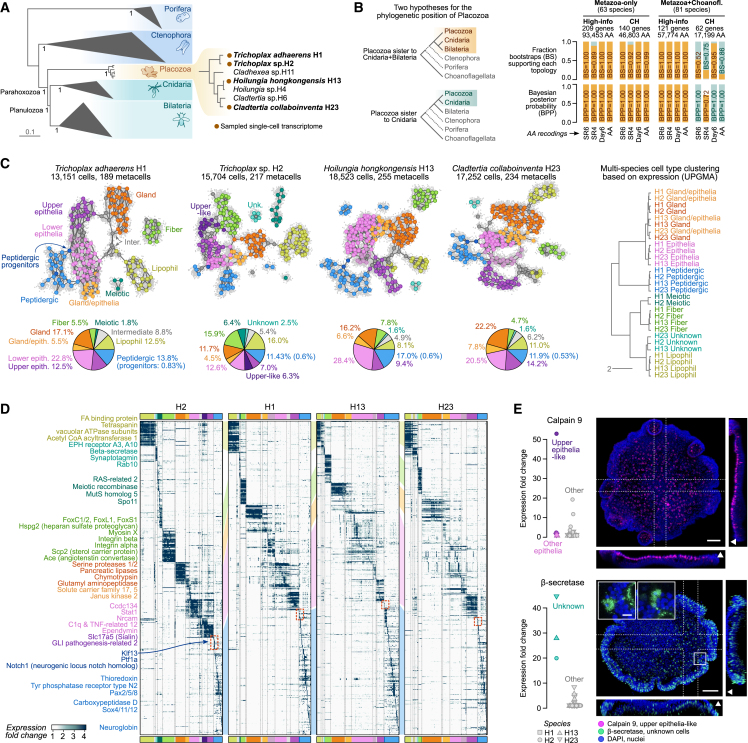
A multi-species placozoan whole-body cell atlas (A) Consensus phylogenetic tree obtained with Bayesian inference under the CAT + GTR + Г4 mixture model on the Metazoa-only 209-markers concatenated aminoacid matrix recoded into 4 categories (SR4). Bayesian posterior probabilities are indicated as supports in key nodes. The cladogram to the right depicts the phylogenetic relationships among placozoans, highlighting the four species here studied. (B) Summary of the statistical support for alternative phylogenetic positions of Placozoa in the different datasets analyzed: (1) only metazoans (63 species) versus metazoans and choanoflagellates as outgroup (81 species); (2) high-information markers (filtered for tree-likeness score with MARE −d 2 parameter) markers filtered for compositional homogeneity (denoted as CH; markers failing the compositional heterogeneity based on simulated alignments using the LG + Γ4 model in p4, at p > 0.01); and (3) original aminoacid multiple sequence alignments versus recoded alignments with three different schemes (SR4, SR6, and Dayhoff6). (C) 2D projection of metacells for each species sampled in this study and pie charts indicating the relative proportion of cells in each broad cell type category, based on a force-directed layout of the metacell co-clustering graph (see STAR Methods). Right, a broad cell type clustering tree of all four species obtained using the UPGMA average algorithm on Log-Det distance matrices, based on binary ortholog activity in each cell type (fold change ≥ 2). (D) Normalized expression of top variable genes (rows, fold change ≥ 2 with a maximum of 15 genes per metacell) across metacells (columns). Broad cell types are color-coded in the x axis and red squares highlight the peptidergic progenitor metacells. (E) Fluorescent HCR-ISH of *Trichoplax* sp. H2 specimens showing the expression of an upper epithelia-like marker (calpain-9, top) and the expression of a marker gene for the unknown cell type (β-secretase, bottom). Images correspond to the maximum projection of 183 and 70 optical sections, respectively. The dotted lines indicate the sections used for the extended orthogonal views (45 slices). Arrowheads in the orthogonal views indicate the upper part of the animals. Insets in the bottom image show the detail of cells localized in the rim of the animal. Cells highlighted in the insets were imaged at higher magnification in the portions indicated with a square. Expression of the marker genes is shown to the left of each panel. Scale bars are 50 μm for the general views and 5 μm for the insets. See also Figures S1, S2, and S3.

**Figure S1:**
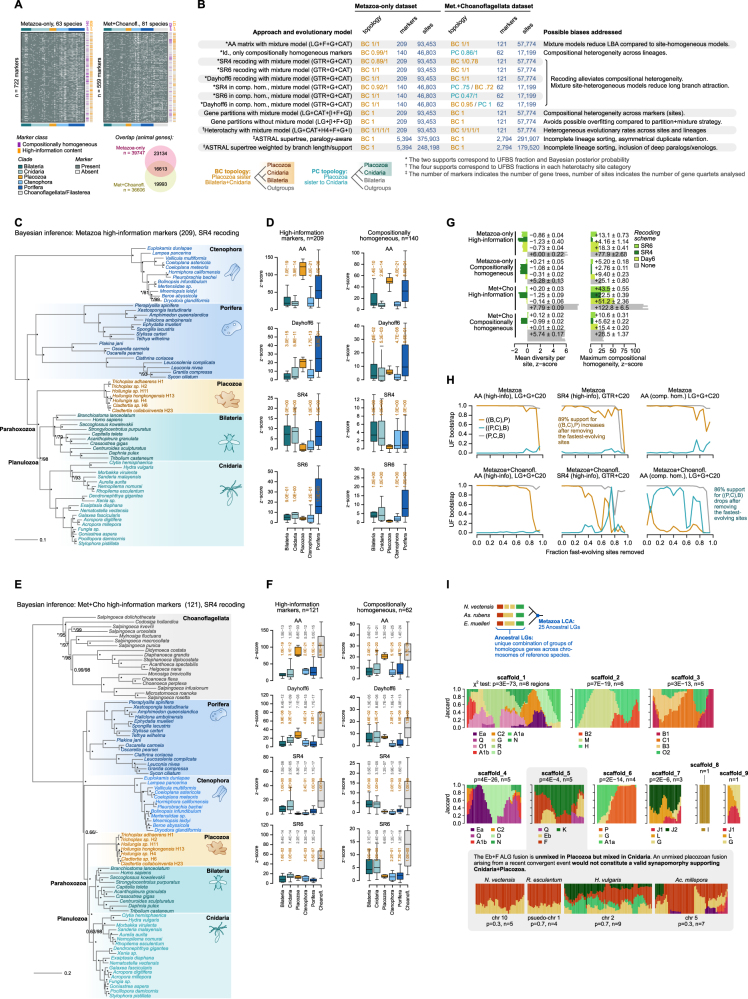
Additional phylogenomic analyses, related to Figure 1 (A) Occupancy matrices for the Metazoa-only (top) and Metazoa + Choanoflagellata datasets (bottom), indicating whether an individual marker (rows) is present or absent in a given species (columns). For each marker, we indicate whether it has been included in the compositionally homogeneous and high-information content subsets. The Venn diagram (bottom) indicates the number of overlapping and exclusive animal genes in both datasets. (B) Summary table of all phylogenetic analyses performed in this study, specifying the topology supported in each analysis (BC, Placozoa sister to Cnidaria and Bilateria; PC, Placozoa sister to Cnidaria) and their statistical support (for ML analyses, fraction of ultrafast bootstrap supports; for Bayesian analyses, Bayesian posterior probabilities or BPP; for super-tree analyses, ASTRAL posterior probabilities). (C) Consensus phylogenetic tree obtained with Bayesian inference under the CAT + GTR + Г4 mixture model on the Metazoa-only concatenated matrix of high-information content markers (n = 209) recoded into 4 categories (SR4). At each node, we indicate Bayesian posterior probabilities and ultrafast bootstrap supports from a ML analysis (C60 + GTR + Г4 mixture model) on the same dataset, with an asterisk denoting full support (100%). (D) Boxplots representing *Z* score values of across-taxa compositional homogeneity tests for individual species, grouped into different clades and under different recoding strategies. We evaluated whether placozoans had higher *Z* score values than other clades with one-sided Wilcoxon tests (p values for each clade above their corresponding boxplots, in orange). (E) Bayesian inference tree obtained using CAT + GTR + Г4 mixture model on the Metazoa + Choanoflagellata concatenated matrix of high-information content markers (n = 121), recoded into 4 categories (SR4). Bayesian posterior probabilities or ultrafast bootstrap supports are indicated at each node, with an asterisk denoting full support (1.0% or 100%, respectively). (F) Same as (C) for the dataset including choanoflagellates as outgroup. Notice in all cases that choanoflagellate *Z* score values are higher than those of other clades, indicating that choanoflagellate empirical aminoacid frequencies strongly deviated from the null posterior predictive distribution. (G) PhyloBayes posterior predictive tests for model adequacy. Left, barplots representing mean *Z* score values of per-site aminoacid diversity tests (PhyloBayes-MPI readpb_mpi—div option) for each of the separate chain run. Right, barplots representing mean *Z* score values of across-taxa compositional homogeneity tests (PhyloBayes-MPI readpb_mpi—comp option) for each of the separate chain run. Numbers indicate mean ± standard deviation of all chains for each dataset. In all cases, the defined burn-ins are the same as for the consensus summary trees (the last 2,000 generations of each chain are employed). (H) Effect of fast-site removal on the support of two phylogenetic hypotheses for Placozoan evolution (sister to Cnidaria and sister to Cnidaria + Bilateria), using the Metazoa and Metazoa + Choanoflagellata datasets. In each case, we removed fast-evolving sites from the high-information markers and reconstructed ML phylogenies with the C20 mixture model. Fast-evolving sites were determined based on their evolutionary rates in the original dataset (per-site rates from IQ-TREE). In all cases, we also display the support for a control, non-controversial node (monophyly of Placozoa, Bilateria and Cnidaria). (I) Ancestral metazoan linkage group signatures along the 9 longest *T. adhaerens* assembly scaffolds (the placozoan with a most-contiguous genome assembly). Using three non-placozoan species with chormosome-scale assemblies as reference (the cnidarian *N. vectensis*, the bilaterian *Asteria rubens* and the sponge *Ephydatia muelleri*), we identified ancestral linkage groups (ALGs) as unique combinations of co-ocurring gene homologs using the same approach as Simakov et al.^27^ Then, we scored the presence of homologs from each ALG along running windows (200 homologous genes, with a 10% steps) in the *T. adhaerens* scaffolds. We find that *T. adhaerens* scaffold 5 contains a partially unmixed fusion of ALGs Eb, F, K, and Q, whereas Eb and F ALGs are fully mixed in cnidarians (*N. vectensis*, *Rhopilema esculentum*, *H. vulgaris*, and *Acropora millepora*), according to χ-square tests of homolog counts for these two ALGs along *n* non-overlapping windows per chromosome.

The phylogenetic position of placozoans is currently debated. Most analyses place Placozoa as the sister outgroup to Cnidaria and Bilateria.^12^^,^^14^^,^^23^^,^^24^ However, a recent study challenged this tree topology, instead suggesting that Placozoa might be a sister to Cnidaria.^25^ Defining the branching position of placozoans in the animal tree is essential to our evolutionary reconstruction, we thus conducted extensive phylogenomic analyses with a sampling of 81 species, including 7 placozoans (Figures 1A and S1A; Table S1). We employed and evaluated 16 datasets (62–209 markers, 17,199–93,453 aminoacid positions) implementing different strategies to minimize compositional heterogeneity across sites and across taxa, such as aminoacid recoding and marker gene filtering with compositional tests (Figures 1B and S1B). These are aimed at ameliorating associated artifacts such as long-branch attraction.^23^^,^^25^ Using PhyloBayes,^26^ we tested the goodness of fit of the CAT + GTR model to these different datasets, concluding that the model adequacy to the data was optimal for datasets excluding choanoflagellates as outgroup and using SR6 or SR4 aminoacid recordings (Figures S1C–S1G). In all these cases, phylogenetic trees consistently supported Placozoa as the outgroup of Planulozoa (Cnidaria + Bilateria) (Figures 1B and S1B). Summary tree and maximum likelihood analyses using both partitioned and unpartitioned mixture models supported the same topology (Figures 1B and S1), whereas the analysis of shared ancient chromosomal linkage groups did not find support for Cnidaria + Placozoa^27^ (Figure S1I). In summary, our analyses suggest that placozoans diverged before the split between cnidarians and bilaterians, and thus, they have a key phylogenetic position to reconstruct early animal evolution.

### A multi-species placozoan whole-body cell atlas

**Figure S2:**
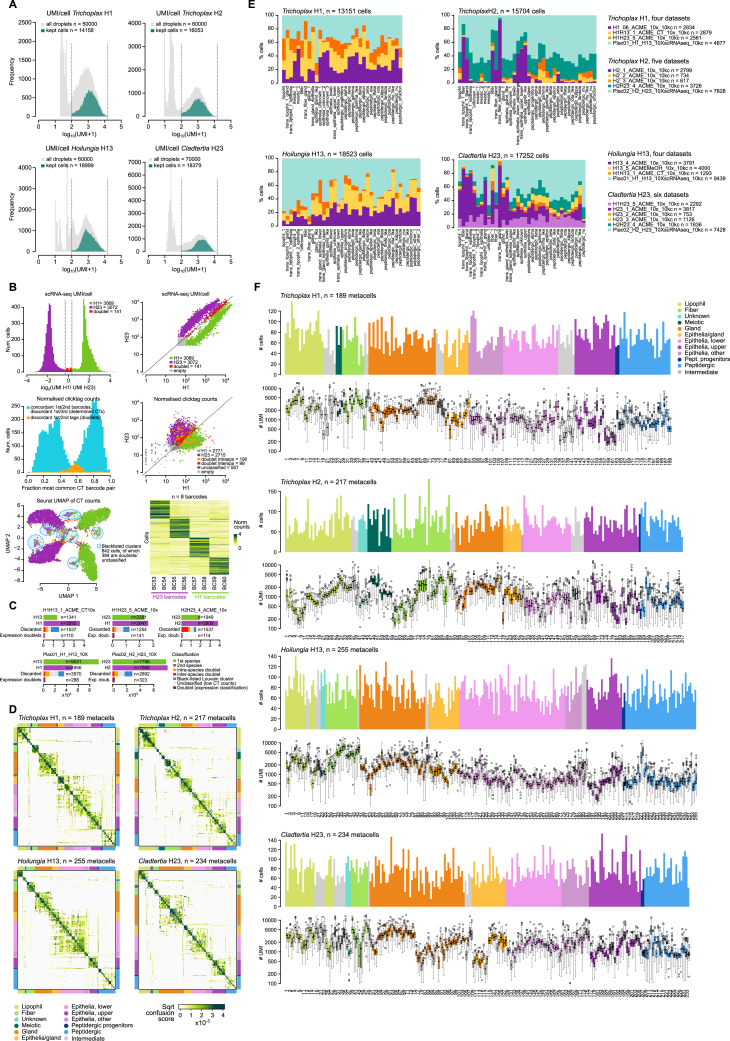
scRNA-seq summary statistics, related to Figure 1 (A) Distribution of total RNA molecules per cell in each placozoan sampled. (B) Clicktag (CT) sample demultiplexing statistics for an example experiment mixing *T. adhaerens* H1 and *C. collaboinventa* H23. Top left: distribution of relative sizes (in UMI/cell) of each cell when their transcriptome is mapped to each of the multiplexed species (*T. adhaerens* H1 and *C. collaboinventa* H23). Top right: UMIs/cell of each cell, classified according to whether its UMI counts are higher in one species or the other, intermediate (doublets), or non-cells (empty droplets). Middle left: fraction of normalized CT counts associated with the most common pair of CT barcodes for each cell, classifying cells in two categories: (1) determined cells, where the first and second most abundant CT barcodes are concordant (from the same sample in the experimental design) but the first and third ones are discordant (from different samples), which represent *bona fide* cells from a single species; or (2) whether the first and second most abundant CTs are discordant (from different samples), which represent possible doublets. Middle right: distribution of CT counts/cell, classified according to whether its CT counts are concordant for one species or the other (determined cells in the left histogram), intra-species doublets (the discordant first and second barcodes come from different samples of the same species), inter-species doublets (the discordant first and second barcodes come from samples of different species), or unclassified (low CT counts). Bottom left: single-cell uniform manifold approximation and projection (UMAP) projection based on normalized CT counts. We removed cells belonging to Louvain clusters with a high fraction of cells classified as doublets in either the cross-species UMI- or CT-based doublet detection procedures (clusters highlighted in blue). Bottom right, heatmap showing the normalized CT counts per single cell (each sample was labeled with two different barcodes, e.g., BC53 + BC54). (C) Summary of the doublet calls for the five CT datasets. Notice the consistency between cross-species UMI- and CT-based doublet calls (which in addition allow us to identify intra-species doublets). (D) Metacell confusion matrices that represent metacell pairwise similarities derived from the K-nn graph connectivity between all cells in each pair of metacells. Colors indicate the broad cell type classification of metacells. (E) Cell type sample composition. (F) Metacell summary statistics. Barplots indicate the number of cells per metacell. Boxplots indicate the number of transcripts/UMIs per single cell grouped into metacells. Colors indicate the broad cell type classification of metacells.

To systematically characterize and compare placozoan cell types, we sampled over 65,000 single-cell transcriptomes from four different placozoans, representing three of the four described genera^12^ (Figures 1C and 1D): *Trichoplax adhaerens* (strain H1, 13,151 cells), *Trichoplax* sp. (H2, 15,704 cells), *Hoilungia hongkongensis* (H13, 18,523 cells), and *Cladtertia collaboinventa* (H23, 17,252 cells). Briefly, specimens were collected, dissociated, and fixed with a modified ACME protocol.^28^ Cells were fluorescence-activated cell sorting (FACS)-sorted to remove doublets, debris, and ambient RNA, before encapsulation and transcriptome capture using 10× Genomics 3′ end single-cell RNA sequencing (scRNA-seq) technology. We sequenced libraries to an average depth of 68,433 reads/cell (mean mappability 64.9%) and obtained a median of 1,759 unique molecular identifiers (UMIs)/cell (Figures S2A–S2C).

We applied the Metacell algorithm^29^ to group cells into transcriptionally coherent clusters (metacells), which constitute our basic unit for downstream analysis. We obtained 189–255 metacells in different placozoans, each metacell containing a median of 70 single cells (Figures S2D–S2F). Based on graph-based 2D projections (Figure 1C) and gene expression patterns (Figure 1D), we identified 28, 29, 32, and 27 cell types/states in H1, H2, H13, and H23 strains, respectively. We further grouped them into nine broad cell types that show relatively similar proportions across the four placozoans (Figure 1C) and that we named according to literature and known markers (Table S2). Of note, we found a cell type exclusive to *Trichoplax* sp. H2 that we termed epithelia upper-like and whose shape and distribution resembles the “concave disks” previously described,^30^ as revealed by hybridization chain reaction fluorescent *in situ* hybridization (HCR-ISH) against calpain-9 (Figure 1E), selected because of its high and specific expression in this cell type. In addition, we applied HCR-ISH against a highly specific β-secretase gene to characterize an unknown type of putatively secretory cells, absent from *T. adhaerens* H1, which localize in the lower part and rim of the animals (Figure 1E).

**Figure S3:**
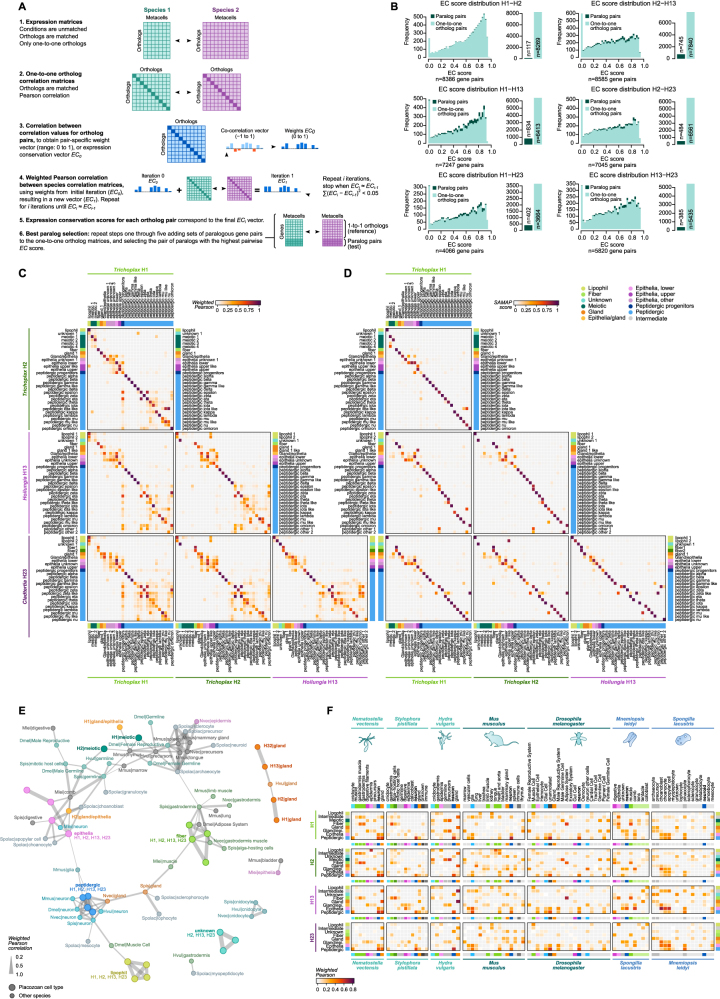
Cell type comparisons across Placozoa, related to Figures 1 and 7 (A) Schematic representation of the main steps in the ICC algorithm applied to metacells. (B) Distribution of ICC-derived expression conservation (EC) scores for each pair of species and for paralog versus ortholog gene pairs. (C) Heatmaps indicating the EC-weighted Pearson correlation between cell types across placozoans. (D) Same as (C) but showing SAMap scores. (E) Force-directed network of cell type similarity across species, using the weighted Fruchterman-Reingold algorithm. Nodes represent cell types (larger nodes correspond to placozoans, smaller ones correspond to other species), and edges represent pairwise similarities as weighted Pearson correlation coefficients. For each cell type, only the top edges are shown (standardized quantile scores above 0.99). Placozoan nodes are color-coded by cell type. Other metazoan nodes are custom color-coded based on similarity to placozoan cell types. (F) Heatmaps representing the transcriptomic similarity between pairs of cell types of the four placozoan species (rows) compared to seven species from other lineages (columns; including three cnidarians, two bilaterians, one sponge, and one ctenophore). Heatmap color reflects the Pearson correlation score between the expression of genes in each cell type (weighting each gene pair with their expression conservation score in that pair of species, using the ICC procedure).

To compare cell type transcriptomes across species, we applied the iterative comparison of co-expression (ICC) algorithm between metacell expression matrices.^31^ Using this method, we defined an expression conservation (EC) score between homologous genes without the need to manually define matching conditions (in this case metacells). This allowed us to select both high EC score orthologs and paralogs across species (Figure S3). In total, we defined a set of 7,389 genes shared across all four placozoans. A broad cell type transcriptome clustering based on these genes revealed strong similarities between the four placozoan species (Figure 1C). Overall, our multi-species single-cell transcriptional atlas represents a comprehensive inventory of the diversity of cell types and cell states in the phylum Placozoa. These atlases can be explored and compared in an interactive database: https://sebelab.crg.eu/placozoa_cell_atlas/

### Intermediate cell states in placozoans

**Figure 2:**
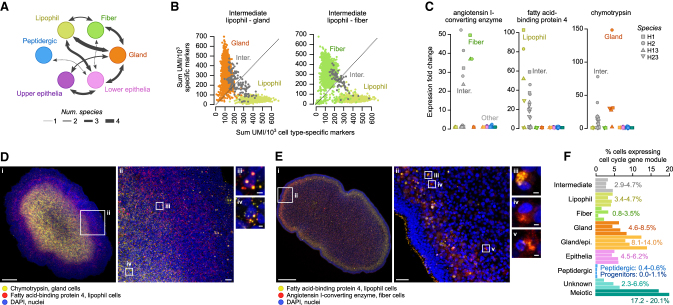
Intermediate cell states in Placozoa (A) Summary of observed intermediate cells between broad cell types. Arrow thickness indicates the number of placozoan species in which we observed the intermediate state. (B) Classification of single cells according to the expression of lipophil-specific gene markers (x axis) and gland- or fiber-specific gene markers (y axis), measured as the fraction of the total UMIs in those cells corresponding to each gene marker list (see all intermediate single-cell profiles in Figure S4C). (C) Expression of fiber (angiotensin I-converting enzyme), lipophil (fatty acid-binding protein 4) and gland (chymotrypsin) gene markers used for HCR-ISH analysis across the four placozoan species. (D) Fluorescent HCR-ISH of *Trichoplax* sp. H2 showing the expression of lipophil (fatty acid-binding protein 4, red) and gland-specific (chymotrypsin, yellow) markers. Images correspond to the maximum projection of 21 (Di) and 55 (Dii) optical sections. Image (Dii) was acquired at higher magnification in the portion indicated with a square in image (Di). Images (Diii) and (Div) show the detail of two cells co-expressing both lipophil and gland-specific markers. Scale bars are 100 μm for the general view (Di), 10 μm for the intermediate view (Dii), and 1 μm for the high magnification images (Diii and Div). (E) Same as (D) for the expression of lipophil (fatty acid-binding protein 4, yellow) and fiber-specific (angiotensin I-converting enzyme, red) markers. Images correspond to the maximum projection of 56 (Ei) and 50 (Eii) optical sections. Images (Eiii), (Eiv), and (Ev) show the detail of three cells co-expressing both lipophil and fiber-specific markers. Scale bars are the same as in (D). (F) Percentage of cells in each major cell type inferred to be in active cell cycle, based on the high expression of S-phase or G2-phase cell cycle gene modules (see Figure 3). See also Figure S4.

**Figure S4:**
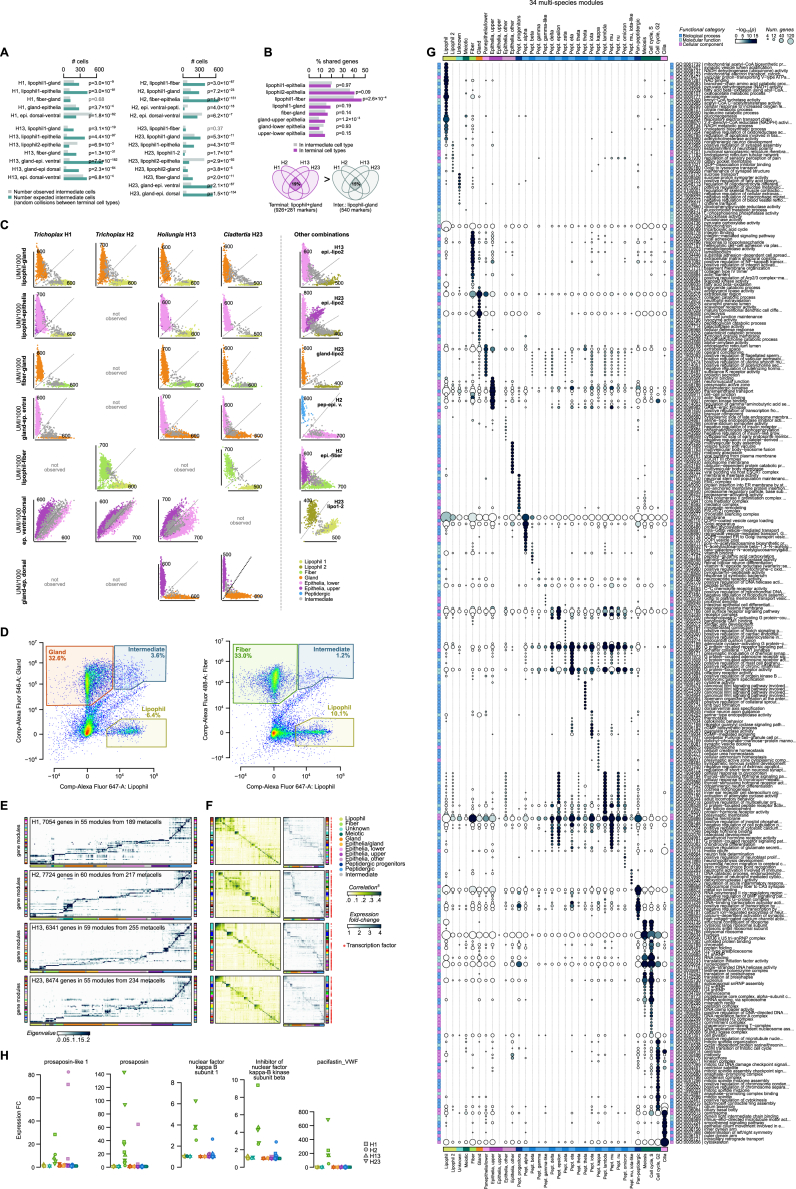
Characterization of intermediate metacells and gene modules, related to Figures 2 and 3 (A) Barplots representing the number of cells classified in each intermediate category (gray) compared with the number of cells doublets in each category (green) that would be expected given the relative frequency of the terminal cell types in each case. We used two-tailed exact binomial tests to determine whether the observed number of intermediate cells significantly differed from the expectation (p values next to each set of bars). (B) Top, barplots representing the number of genes shared in intermediate metacells between the placozoan species where each cell type is found (gray) compared with the number of genes shared by the respective terminal cell types (green). We used one-tailed exact binomial tests to determine whether the number of genes shared across species was higher for terminal than for intermediate cell types (p values shown for each cell type). Notice that in most cases the difference is small and non-significant, indicating that the genes expressed in intermediate cells are conserved across species and not a stochastic sampling of genes expressed in the respective terminal cell types. Bottom, Venn diagrams detailing the number of shared genes across species for lipophil-1/gland intermediate cells (gray) compared with the shared genes by lipophil-1 and gland cell types (green). (C) Intermediate cells exhibit intermediate transcriptional signatures between their terminal cell types. For each pair of cell type in each species, we show the sum of the fraction of UMIs (per 1,000 UMIs) of the top markers (FC ≥ 2). Panels are arranged to indicate the detection of specific intermediate cell types (rows) in each of the species (columns). (D) Flow cytometry scatterplots of *Trichoplax* sp. H2 cells labeled by HCR-ISH against markers specific for lipophil (fatty acid-binding protein 4, Alexa Fluor-647), gland (chymotrypsin, Alexa Fluor-546) and fiber (angiotensin I-converting enzyme, Alexa Fluor-488) cells. Selected areas in each panel denote the percentage of cells with single or double label, which would correspond to intermediate cells. (E) Heatmaps representing the eigengenes across metacells of gene modules calculated using WGCNA in each placozoan. x axis colors indicate the broad cell type classification of metacells. Module colors (y axis) are arbitrary. (F) Left, gene-gene expression correlation matrix, grouping genes into the same modules as in (E). Right, normalized expression across metacells of genes grouped into modules. Transcription factors are highlighted with a dot to the right of the heatmap. Notice the presence of “lateral” gene modules expressed in individual metacells across cell types. These include, for example, the cell cycle and ciliary apparatus modules. (G) Top 10 gene ontology terms enriched in each multi-species gene module. x axis colors indicate the cell type where each module is most active (manual curation). (H) Fold-change expression of selected genes with immune-related functions across cell types of all four placozoans.

Upon examination of metacell gene expression maps and confusion matrices (Figure S2D), we detected the presence of metacells with transversal expression profiles between cell types, which we termed “intermediate” cells (Figures 2A, 2B, and S4). Intermediate cells appeared in all four species, and statistical tests showed that intermediate cell occurrence cannot be explained by cell doublets arising from stochastic co-encapsulation (Figure S4A); for example, peptidergic cells are seldom involved in intermediate metacells. Intermediate cells lack specific gene markers, and they only expressed a small subset of the genes expressed in each of the terminal cell types, for example, *T. adhaerens* H1 lipophil-gland intermediate cells express 33% of lipophil markers and 10% of gland cell markers (mean of 33% and 13% in other species). Moreover, this subset of expressed genes was not random, instead showing a degree of conservation across species similar to that observed between terminal cell types (Figure S4B). Using HCR-ISH, we could confirm the presence of cells co-expressing both lipophil- and gland-specific markers (Figures 2C and S4D): fatty acid-binding protein 4 and chymotrypsin, respectively (Figures 2D and S4D). Similarly, we could identify cells co-expressing the same lipophil-specific marker and the fiber-specific marker angiotensin I-converting enzyme (Figures 2C, 2E, and S4D). Finally, we observed that 4%–14% of the terminally differentiated cells in placozoans expressed cell cycle genes—again with the notable exception of peptidergic cells (Figure 2F). The presence of dividing and potentially transdifferentiating adult cell types adds to similar evidence in other non-bilaterians like sponges^32^ and suggests that plastic cell fates might have been common in early animal evolution.

### Placozoan functional gene modules

**Figure 3:**
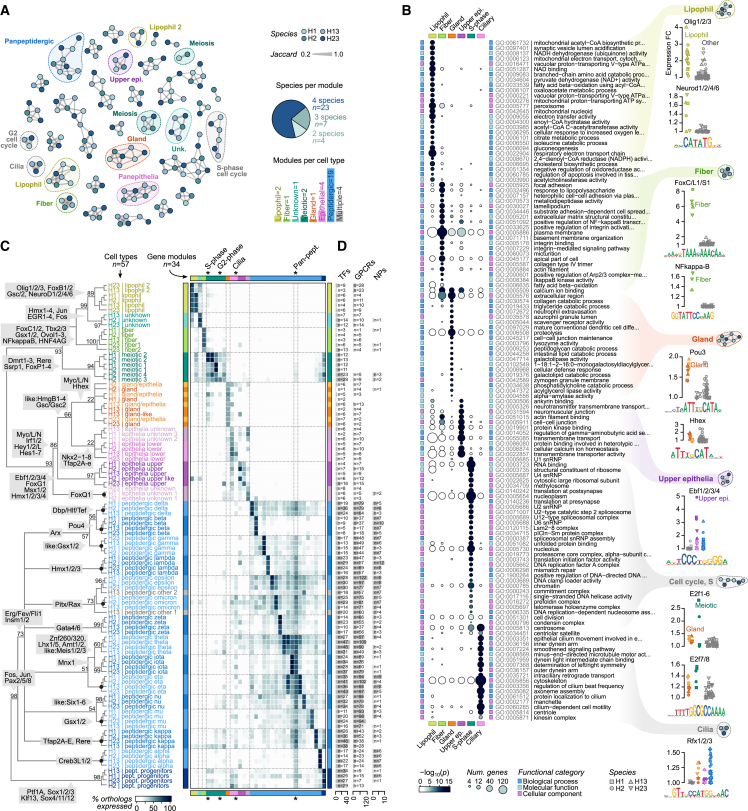
Placozoan gene expression programs (A) Multi-species clustering of gene modules across placozoans. Each node represents a gene module (group of genes co-expressed across metacells; see Figures S4E–S4G), and each node is color-coded according to the species. Edges link modules sharing orthologs across species, and their width reflects the Jaccard index of ortholog overlap between modules (only edges with Jaccard ≥0.125 are shown). We curated 34 multi-species modules, the majority of which are composed of modules from four species (pie plot). Most modules are specific to individual cell types (bar plot), with the exception of cross-cell type modules that include genes related to pan-peptidergic cells, cell cycle (S-phase and G2-phase), meiosis, and the ciliary apparatus. (B) Gene ontology enrichments in selected gene modules (left), and expression of transcription factor (TF) regulators and associated enriched motifs (right). (C) Left, multi-species clustering of non-peptidergic (top) and peptidergic cell types (bottom). The cell type tree has been obtained as in Figure 1C. Gray boxes list selected TFs specific to various cell type clades. Right, heatmap depicting the fraction of orthologous genes from each gene module expressed across cell types. Modules have been color-coded according to their cell type specificity, with the cross-cell type modules highlighted with asterisks. (D) Number of TFs, GPCRs, and neuropeptides (NPs) expressed (fold change ≥ 2) in each cell type. See also Figures S4, S5, and S6.

To understand the structure and evolution of placozoan gene expression programs, we clustered genes into modules based on metacell co-expression in each of the species. These gene modules recapitulated cell-type-specific expression programs, but we also identified cross-cell type functional modules (Figures S4E–S4G). Comparative analysis revealed 34 highly conserved gene modules across species (Figures 3A–3C; Table S3), including two cell cycle modules (representing S-phase and G2-phase), a meiosis/gametogenesis module, a ciliary module, a pan-peptidergic module, and gene modules associated to the major somatic cell types (upper and lower epithelial cells, gland cells, lipophil cells, and fiber cells). For example, the fiber cell module includes genes involved in actomyosin contractility, integrin cell adhesion, and lamellipodia formation, as well as bacterial response genes and the immune transcription factor (TF) nuclear factor (NF)-κB (Figures 3B and S4H). This is in line with a potential role for fiber cells in placozoan immunity.^19^ The lipophil cell modules includes genes involved in fatty acid and cholesterol metabolism, as well as in aminoacid catabolism associated with energy production (Figure 3B), while the gland cell module is characterized by protein, lipid, and carbohydrate degradation processes (Figure 3B).

**Figure S5:**
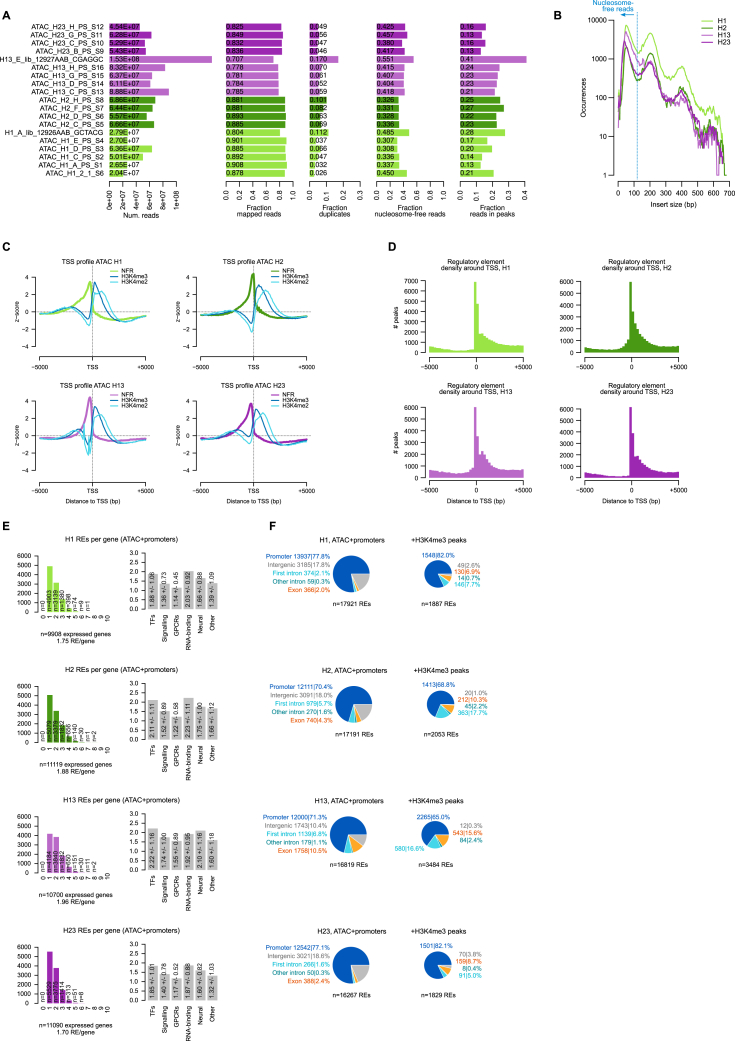
Placozoa chromatin landscapes, related to Figure 3 (A) Summary statistics of ATAC experiments. From left to right: number of reads, fraction of reads mapped in the genome, fraction of duplicated reads (based on mapping coordinates of read pairs), fraction of nucleosome-free reads (that are used for *cis*-regulatory element/peak calling), and fraction of reads in peaks. (B) ATAC-seq fragment size distribution. The line indicates the cutoff used to define nucleosome-free reads. (C) Transcription start site (TSS) metaplots for ATAC-seq nucleosome-free reads (NFRs) and H3K4me2/H3K4me3 ChIP-seq signal. (D) Frequency of regulatory elements (REs) around the TSS. (E) Distribution of number of REs per gene (left), and the average number of REs in various gene categories (right; values indicated a mean ± standard deviation). (F) Association of ATAC-seq peaks and H3K4me3 ChIP-seq peaks with different genome-wide features.

**Figure S6:**
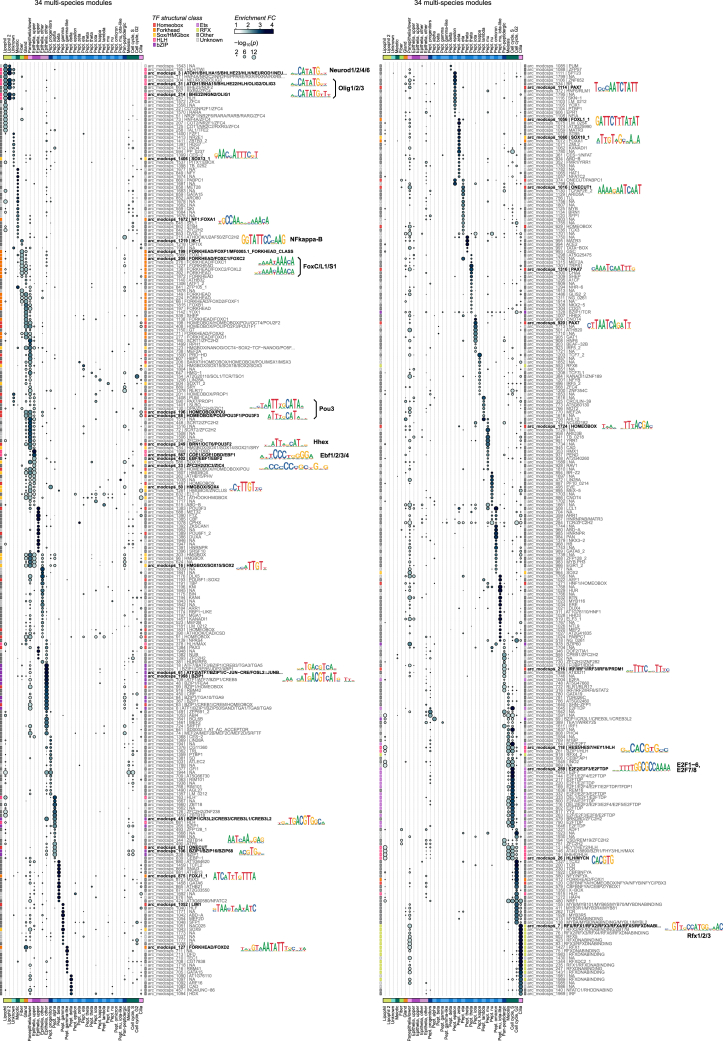
Transcription factor binding motif analysis, related to Figure 3 Motif archetype enrichment in the REs associated to genes belonging to each of the 34 multi-species gene modules. Dot color indicates the intensity of the enrichment fold change between the counts of each motif in that gene module’s genes (including only motifs with an alignment score higher than the 98th quantile of their genome-wide alignment score distribution), and using genes associated to other modules as background; dot size indicates the p value of a hypergeometric enrichment test, adjusted using a false discovery rate. Up to 20 marker archetypes with FC ≥ 1.5 are shown per module. The structural class of each motif archetype, as inferred from known motifs similar to it, is shown next to each motif (colored squares). Selected motif archetypes (scaled to information content) are shown next to the heatmap. The motifs labeled with gene names (Neurod1/2/4/6, Olig1/2/3, NF-κB, FoxC/L1/S1, Pou3, Hhex, E2F1–6, and E2F7/8) correspond to genes shown in Figure 3B.

To further dissect the regulation of these modules, we generated assay for transposase-accessible chromatin with sequencing (ATAC-seq) and H3K4me2/me3 chromatin immunoprecipitation sequencing (ChIP-seq) data to annotate *cis*-REs genome-wide in each species (Figures S5A–S5C). We identified 19,286–25,164 REs genome-wide in the four placozoans, mostly located proximally to the promoter and in the first introns (Figures S5D–S5F). Using these RE maps, we then surveyed the TF-binding motifs enriched in REs associated with the genes from each module. First, we performed *de novo* motif enrichment and then combined its results with the known, experimentally determined motifs, based on sequence similarity, to create a consensus set of motif archetypes. Enrichment analysis using these motif archetypes revealed that the regulatory signatures linked to the different gene modules (Figures 3B and S6), with frequent cases of coinciding expression and motif enrichment for a particular TF. These analyses revealed the lexicon of motifs associated with placozoan cell identities.

### Genetic basis of placozoan cell type evolution

**Figure 4:**
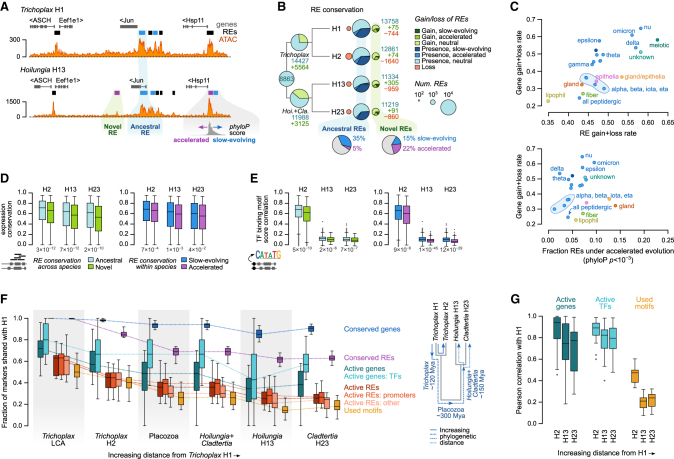
Genetic basis of cell type evolution in Placozoa (A) Aligned genomic region exemplifying different categories of regulatory element (RE) conservation. Each RE is classified according to two criteria: across-species conservation (ancestral/novel) and intra-species sequence dynamics (conserved/neutral/accelerated). (B) Ancestral reconstruction of RE evolution across Placozoa. In extant nodes, REs are classified according to their sequence conservation/acceleration status. (C) Rates of evolution in the transcriptional and regulatory profiles of matched cell types across all four placozoans. For each cell type, we recorded the fraction of specific markers (genes expressed at FC ≥ 1.5) that were gained or lost at least once along the placozoan phylogeny (y axis) and compared them (x axis) with the rate of active RE gain + loss along the same branches (top) or to the fraction of active REs that exhibited signatures of accelerated evolution (x axis, bottom, at *phyloP* < 0.001) in extant species (bottom). (D) The impact of RE sequence dynamics in gene expression conservation, comparing *Trichoplax adhaerens* H1 to the other three placozoans. Left, boxplot comparing the expression conservation score of orthologous genes with shared ancestral REs to those of genes with novel REs. Right, boxplot comparing the expression conservation of orthologs with slow-evolving REs to orthologs with one or more accelerated RE. We used one-sided Wilcoxon rank sum tests to test for significant differences in the EC score distributions (p values below each pair of boxplots). (E) Same as (D) but comparing TF-binding motif usage similarity (Spearman correlation of gene-wise maximum motif alignment score). (F) Evolutionary dynamics of various genetic determinants of cell identity at increasing evolutionary distances. The boxplot represents the fraction of shared features for each matched cell type and from the perspective of *T. adhaerens* H1. Compared features include: conserved genes (genes expressed in a given *T. adhaerens* cell type with an ortholog in the genome of the other species or reconstructed ancestor), conserved REs (likewise, using sequence conservation of orthologous REs), active genes (genes expressed in a given cell type in both *T. adhaerens* H1 and the other species), active REs (REs linked to genes expressed in a given cell type in both *T. adhaerens* H1 and the other species), and used motifs (TF-binding motifs enriched in both *T. adhaerens* H1 and the other species). The cladogram shows the time-calibrated distances.^12^ (G) Distribution of the correlations in gene expression and TF-binding motifs across cell types (both measured as fold-change enrichments) between *T. adhaerens* H1 and the three other placozoans.

Our multi-placozoan cell atlas and chromatin maps offer the opportunity to study the genetic determinants of cell identity and their evolutionary dynamics within an entire animal phylum: from genome sequence through RE usage to the observed gene expression phenotype. To this end, we classified RE sequence conservation at two levels (Figure 4A): (1) across-species conservation (ancestral/novel) based on whole-genome alignments and (2) intra-species sequence dynamics (slow-evolving/neutral/accelerated) according to *phyloP* scores^33^ calculated using 4-fold degenerate codon sites as a background model.

Reconstruction of RE evolutionary dynamics across Placozoa reveals nearly 9,000 ancestrally conserved REs and thousands of gains in the H1 + H2 (5,564 REs) and H13 + H23 (3,125 REs) ancestors, followed by rather modest gains in each of the extant placozoans (Figure 4B). As expected, among the taxon-specific REs, we detect an over-representation of REs with signatures of accelerated evolution. Reciprocally, among ancestral REs, there is an over-representation of slow-evolving elements with signatures of stabilizing selection (Figure 4B). When stratified by cell type, we observe that some cells like lipophil and fiber show slower rates of RE gains and losses (Figure 4C, top) and lower proportion of REs under accelerated evolution (Figure 4C, bottom), a trend that is mirrored by the rates of gene gains and losses (Figure 4C).

We then interrogated the impact of RE sequence dynamics on gene expression evolution (Figure 4D), using *T. adhaerens* H1 as a viewpoint. In all comparisons, the presence of an ancestrally conserved RE between a pair of homologs was associated with significantly higher EC. Also, the presence of a RE with a signature of accelerated sequence evolution was linked to lower EC between genes (Figure 4D). A similar pattern was observed for the conservation of TF-binding motifs (Figure 4E).

Finally, we examined the degree of conservation of different genetic determinants of cell identity between matched cell types at different phylogenetic distances. Specifically, we measured the fraction of genes, REs, and TF-binding motifs shared between one reference species (*T. adhaerens* H1) and other placozoans, including reconstructed ancestors (Figure 4F). TF usage was the most conserved feature across cell types, followed by effector gene usage. The REs linked to these shared genes show a lower degree of conservation, particularly non-promoter elements. Finally, TF-binding motif usage was the most rapidly diverging of all features (Figure 4F). The relative conservation of gene expression (higher) and motif enrichments (lower) was also apparent when measured using Pearson correlation coefficients in extant species, again from the H1 viewpoint (Figure 4G). The evolutionary conservation of these cell identity characters across placozoans is similar to the estimated conservation between mammals and between insects.^34^^,^^35^

### Diversity of peptidergic cell types

**Figure 5:**
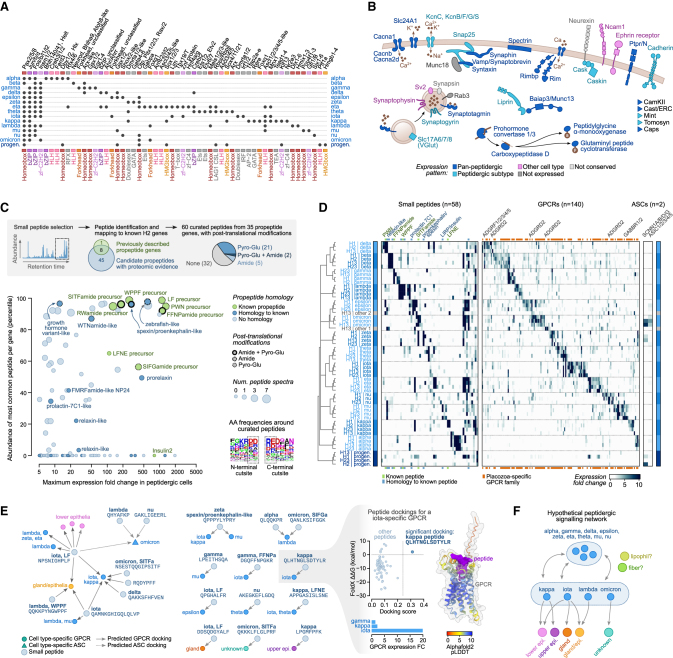
Diversity of peptidergic cell types in placozoans (A) Combinatorial expression of TFs across four placozoans. Dots indicate that a given TF has been inferred to be specifically expressed (FC ≥ 1.5) in a given peptidergic type at the last common ancestor of placozoans (based on Dollo parsimony). (B) Schematic representation of the pre-synaptic scaffold components expressed in placozoan peptidergic cells. Individual gene expression plots for the four species are shown in Figure S7. (C) Identification of *Trichoplax* sp. H2 small peptides. Scatter plot shows the maximum expression of the propeptide gene in any peptidergic cell type (x axis) compared with the abundance of the most common peptide per propeptide as measured by mass spectrometry (y axis). Dot sizes indicate the number of spectra identified for the most common peptide per propeptide. The color code indicates homology of the propeptide and dot border lines indicate the identification of peptide post-translational modifications. Motifs represent aminoacid frequencies around peptides. (D) Combinatorial expression of neuropeptides (NPs) and their putative receptor gene families (GPCRs and amiloride-sensitive channels [ASCs]) in peptidergic cell types across four placozoans. In the NP map, known peptides (green) or new, hypothetical peptides with homology to previously described NPs (blue) are indicated. In the GPCR map, genes with no orthologs in other animal phyla (i.e., Placozoa-specific families) are indicated (orange), whereas known families are indicated by name. (E) Network of hypothetical interactions between cell-type-specific small peptides and receptors (GPCRs and ASCs). Gray nodes indicate small peptides with an indication of the aminoacid sequence and of peptidergic cell type expressing their propeptide. The colored nodes represent receptors (GPCRs as circles and ASCs as triangles), and are color-coded according to their cell type specificity. Arrows represent hypothetical compatible interactions between NPs and receptors based on the joint three-dimensional modeling of the docked peptides for all NP-receptor combinations. In brief, we have considered all interactions with high docking scores (pDockQ > 0.23) and a positive change in ΔΔG values between the wild-type and a mutated version of the NP (FoldX ΔΔG > 0 kcal/mol; see STAR Methods for details and Table S5 and Figure S7E for a complete list of all positive interactions). An example model of a positive docking is shown at the right, including its AlphaFold prediction (receptor residues colored according to the model accuracy using the predicted local distance difference test or pLDDT score). (F) Schematic hypothetical network of cell type signaling interactions based on the inferred NP-receptor pairs from (E). This is a hypothetical model based on predicted affinities between a partial set of NPs and only a subset of all hypothetical receptors (cell-type-specific GPCRs and ASCs), and is therefore partial, leaving certain cell types unconnected (e.g., fiber and lipophil). See also Figure S7.

In all four cell atlases, we identified a high diversity of peptidergic cells. The cross-species analysis allowed us to group them into fourteen types (Figures 3C, S3C, and S3D), which we denote with Greek letters (alpha to omicron). These types represented two-thirds of the somatic cell types in spite of being only 11%–17% of the single cells sampled in each taxon. All peptidergic cells expressed a high number of specific TFs (median 34, compared with 6 in others; fold change [FC] ≥ 2) (Figure 3D) and in unique combinations (Figure 5A) that always included a distinct homeobox TF (Figure 5A). Similarly, peptidergic cell types expressed a high number of G protein-coupled receptors (GPCRs,median 25, compared with 2 in others) in unique combinations (Figures 3D and 5D). These GPCRs largely represent a placozoan-specific expansion, with no orthologs in other animal phyla (Figure 5D; Table S4).

**Figure S7:**
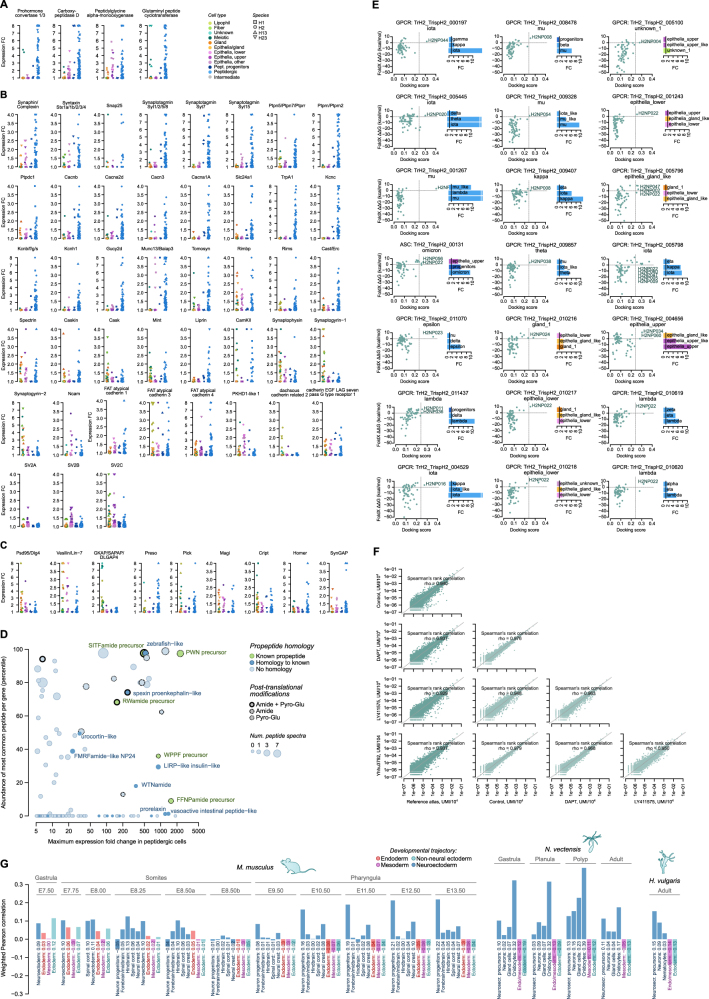
Peptidergic cell transcriptional profiles, related to Figures 5 and 6 (A) Expression fold change (FC) of neuropeptide-processing enzymes across species and cell types. Cell types are grouped in four categories, from right to left: peptidergic (light blue), peptidergic progenitors (dark blue), epithelial, and others. Species are indicated with different shapes. (B) Same as (A) for pre-synaptic scaffold genes. (C) Same as (A), for post-synaptic scaffold genes. (D) Identification of *H. hongkongensis* H13 small peptides. Scatterplot shows the maximum expression of the propeptide gene in any peptidergic cell type (x axis) compared to the abundance of the most common peptide per propeptide as measured by mass spectrometry (y axis). Dot sizes indicate the number of spectra identified for the most common peptide per propeptide. The color code indicates homology of the propeptide and dot border lines indicate the identification of peptide post-translational modifications. (E) Scatterplots showing the two docking scoring metrics (see STAR Methods) for positive docking peptide receptor pairs shown in Figure 5E. Barplots on the right show the expression pattern for the corresponding receptor (only for cell types with FC ≥ 1.5). (F) Comparison of global gene expression levels for the three *Trichoplax* sp. H2 Notch signaling drug treatments (plus DMSO control sample) compared with the reference *Trichoplax* sp. H2. Scatterplots show the normalized UMI counts per gene in each sample. The Spearman correlation for each comparison is indicated. (G) Expression similarity between placozoan peptidergic progenitor cells (H2 pooled dataset) and cell types assigned to various developmental trajectories in other species (mouse, *N. vectensis* and *H. vulgaris*), measured as weighted Pearson correlation coefficients of cell type-level FC values. Gene markers were selected from ICC-defined ortholog pairs belonging to predicted transcriptional regulator gene families (transcription factors, chromatin regulators, and RNA-binding proteins), and we restricted the analysis to genes with variable expression in both datasets (FC ≥ 1.25 in at least one cell type in both the placozoan reference and the query dataset, totaling 73–207 genes in mouse, 172–367 in *N. vectensis*, and 318 in *H. vulgaris*). For each developmental trajectory (the various neuroectodermal lineages shown in Figure 6H, plus endoderm, mesoderm, non-neural ectoderm, and endo/mesoderm), we report the the correlation with the most similar cell type in each combination of developmental stage and lineage.

Peptidergic cells share a common gene module that includes the TFs Pax2/5/8, Jun, and Fos (Figure 5A); the four enzymes involved in NP-processing steps (prohormone convertase, two carboxypeptidases, peptidylglycine alpha-amidating monooxygenase, and glutaminyl peptide cyclotransferase) (Figures 5B and S7A); the proton-based glucose transporter Slc45; the glutamate transporter Slc17a; the RNA-binding protein Cpeb, which regulates RNA polyadenylation in bilaterian neurons; and the Rho guanine exchange factor Kalirin, involved in neuronal morphogenesis in bilaterians. In addition, peptidergic cells expressed the most known genes conforming to the pre-synaptic scaffold in cnidarian and bilaterian neurons^36^ (Figures 5B and S7B). These include proteins involved in neurosecretory vesicles (Syntaxin, Synaphin, Snap25, Unc13, and Tomosyn), active zone proteins (Rims and Caskin), and specific calcium voltage-gated channels and auxiliary subunits that mediate vesicle release. In contrast, we did not detect any co-expression of known components of the post-synaptic scaffold in peptidergic or any other cell type (Figure S7C).

Next, we sought to determine the repertoire of secreted peptides expressed in the fourteen identified peptidergic cell types. To this end, we performed mass spectrometry-based peptide identification for *Trichoplax* sp. H2 and for *H. hongkongensis* H13 (Figures 5C and S7D; Table S5). We identified 60 small peptides corresponding to 53 peptide precursor genes, expanding the repertoire of previously predicted peptides^21^^,^^22^^,^^37^ (Figure 5C). Half of the identified peptides showed evidence of N-terminal pyroglutamination and/or C-terminal amidation (Figure 5C), two post-translational modifications typically associated with NPs in other species.^38^ The genes encoding for these peptides are highly expressed (Figure 5C) in specific peptidergic cell types (Figure 5D). Next, we predicted the hypothetical receptors of these experimentally identified peptides. Most animal NPs bind to GPCRs^39^ and a few also to amiloride-sensitive channels (ASCs).^40^ Thus, we selected *Trichoplax* sp. H2 expressed GPCRs and ASCs (196 and 11 in total, respectively), and we used AlphaFold2^41^^,^^42^ to jointly model the structure of each of these candidate receptors with each of the peptides.^43^ High-scoring structural models were selected and we further measured docking between the peptide and the receptor using two independent metrics. This analysis identified 30 receptor-peptide pairs with high docking scores and specificity (of the 21 significant receptors, 17 have only one specific peptide) (Figures 5E and S7E). Combined with the expression pattern of the peptide precursors (emitting cells) and the expression of receptors in other cells, we inferred a hypothetical peptidergic cell signaling network in *Trichoplax* sp. H2 (Figure 5F). Overall, the diversity of peptidergic cell types and NPs we identified indicate the potential for highly complex paracrine signaling in placozoans.

### Peptidergic cell progenitors with neurogenesis signatures

**Figure 6:**
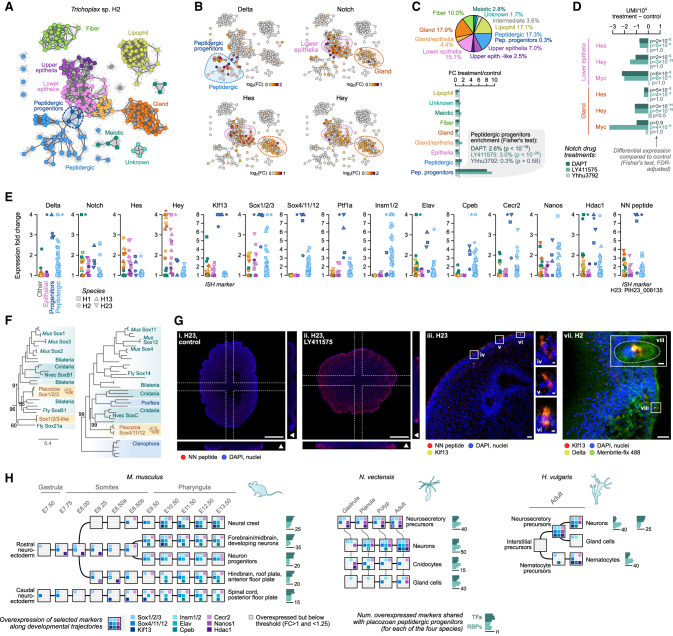
Molecular signatures of neurogenesis in placozoans peptidergic cell progenitors (A) 2D projection of metacells of a *Trichoplax* sp. H2 single-cell pooled transcriptome of individuals grown under four conditions: treatment with the Notch antagonists DAPT (3,453 cells) and LY411575 (4,666 cells), the Notch signaling agonist Yhhu3792 (5,114 cells), and an untreated control (4,765 cells). Metacells have been color-coded by broad cell type based on comparison to the reference *Trichoplax* sp. H2 dataset (Figure 1C). (B) Normalized expression of Delta, Notch, Hes, and Hey in the 2D projection of *Trichoplax* sp. H2 metacells. (C) Pie plot with cell type proportions among the control cells (top) and fold-change enrichment in cell type fractions for each drug treatment (bottom). p values from a two-sided Fisher’s exact test of cell type counts relative to the control. (D) Differential expression of the Hes, Hey, and Myc TFs in lower epithelial and gland cells (two cell types with broad Notch expression), measured using the difference in UMIs/10^4^ between treatment and control. p values indicate significant differential expression based on a two-sided Fisher’s exact test on UMI counts. (E) Expression of selected marker genes related to peptidergic progenitor specification across all four placozoans, including markers used for HCR-ISH experiments. p values from an FDR-adjusted two-sided Fisher’s exact test of UMI counts in a given cell type, relative to the control. (F) Sox TF maximum likelihood phylogenetic analysis supporting the orthology of placozoan Sox1/2/3 and Sox4/11/12. (G) Left, fluorescent HCR-ISH of *C. collaboinventa* H23 showing the expression of the peptidergic progenitor-specific marker HoiH23_PlH23_008135 (NN peptide, red) in animals with (Gii) and without (Gi) treatment with 10 μM LY411575 for 24 h. Images are maximum projections of 50 (Gi) and 40 (Gii) optical sections. The dotted lines indicate the sections used for the extended orthogonal views (40 slices). Arrowheads in the orthogonal projections indicate the upper part of the animals. Middle, fluorescent HCR-ISH of *C. collaboinventa* H23 (Giii and Gvi) showing the expression of the peptidergic progenitor-specific markers HoiH23_PlH23_008135 (NN peptide, red) and Klf13 (yellow). Image (Giii) is a maximum projection of 22 optical sections. Images (Giv) to (Gvi) highlight the detail of three individual cells expressing both markers and correspond to the squared sections of image (Giii). Right, fluorescent HCR-ISH of *Trichoplax* sp. H2 (Gvii and Gviii) showing the expression of the peptidergic progenitor-specific markers Klf13 (red) and Delta receptor (yellow). Image (Gvii) is a maximum projection of 16 optical sections. Inset (Gviii) highlights the detail of a cell expressing both markers. Dotted line depicts the shape of the cell as delineated by the membrane marker (green). Scale bars correspond to 100 μm in i and ii, 10 μm in (Giii) and (Gviii), and 1 μm for (Giv)–(Gvi) and (Gviii). (H) Expression of selected TFs, RNA-binding proteins and chromatin factors specific to placozoan peptidergic progenitors (E) along the neural developmental trajectories described in scRNA-seq experiments in *M. musculus* (gastrula to pharyngula stage^44^), *N. vectensis* (gastrula to adult^45^), and *Hydra vulgaris* (regenerating adult^46^). Genes with expression FC ≥ 1.25 in any cell type of a given developmental trajectory are indicated as colored squares in each (overexpressed genes with FC > 1 and < 1.25 in stages intermediate between two other stages are indicated with a white asterisk). For each developmental trajectory, we also indicate the number of orthologous TFs and RBPs shared with each placozoan species (barplots to the right). See also Figure S7.

We also identified in all four species a population of peptidergic progenitors that shared some transcriptional similarities with lower epithelial cells (Figure 6A) and showed no gene expression signatures of cell division (Figure 2F). These lower epithelial cells, as well as gland cells, broadly expressed Notch receptors and their downstream repressor TFs (Hes and Hey) (Figures 6B and 6E). In contrast, the peptidergic progenitors expressed both Notch and Delta receptors (Figures 6B and 6E). In different species, Delta-Notch lateral inhibition within an epithelium distinguishes one cell type from another, particularly neuronal from non-neuronal cells.^47^ Thus, this might represent the induction signal for lower epithelial cells to differentiate into peptidergic cells. To further explore this hypothesis, we treated *Trichoplax* sp. H2 animals with two chemical Notch antagonists (DAPT^48^ and LY411575^49^) and one agonist (Yhhu-3792^50^), and together with a control sample, we jointly profiled them in a scRNA-seq experiment (Figures 6A and S7F). These antagonists have been shown to cause an increase in neurogenesis and a downregulation of Hes in the cnidarian *Nematostella vectensis*^51^^,^^52^^,^^53^ and other species.^54^^,^^55^ Both Notch antagonists produced a significant increase in the frequency of peptidergic progenitor cells in *Trichoplax* sp. H2 (Figure 6C), whereas the agonist did not cause any effect. Moreover, the Notch antagonists caused a significant decrease in the expression of the TFs Hes and Hey in the Notch receptor-expressing cells (Figure 6D). We also observed in epithelial and gland cells (which both show signatures of cell division, see Figure 2F) a decrease in the expression of the cell proliferation-associated TF Myc. A link between Notch signaling and proliferation has been also suggested in other species.^55^^,^^56^ The effect of the Notch antagonist LY411575 increasing the frequency of peptidergic progenitor cells was also observed in another placozoan species, *C. collaboinventa* H23, using HCR-ISH against an unknown protein coding gene HoiH23_PIH23_008135 (Figure 6G), selected because its high and specific expression in progenitor cells (Figure 6E).

We then examined the expression of specific markers in peptidergic progenitor cells. This included chromatin remodelers and modifiers (Hdac1, Smarca1, Brd1, and Cecr2), diverse RNA-binding proteins (e.g., Elav, Nanos, and Cpeb), and the TFs Ptf1a,^57^^,^^58^^,^^59^ Sox1/2/3 (SoxB), Sox4/11/12 (SoxC), Klf13,^60^^,^^61^ and Hbp1^62^^,^^63^ (Figures 6E and 6F). Coinciding with the expression of Sox1/2/3 and Sox4/11/12, Sox TF-binding motifs are also strongly enriched in the *cis*-regulatory regions of genes expressed in progenitor cells (Figure S6). To characterize the location and morphology of peptidergic progenitor cells, we performed multiplexed HCR-ISH against Klf13 together with HoiH23_PIH23_008135 in *C. collaboinventa* H23 (Figure 6G) or Klf13 and the Delta receptor in *Trichoplax* sp. H2 (Figure 6G). Peptidergic progenitors localized at the rim of the animals and their elongated morphology resembles that of columnar epithelial cells. Finally, when examining developmental scRNA-seq from other species (Figures 6H and S7G; Table S3), we detected conserved expression of some of these peptidergic progenitor markers in neuronal/neurosecretory precursors, including RNA-binding proteins (RBPs) and TFs like Sox1/2/3 and Sox4/11/12 (Figure 6H). Overall, the molecular signatures identified in peptidergic progenitors are intriguingly similar to those in neuronal progenitors in cnidarians and bilaterians.^45^^,^^47^^,^^64^^,^^65^

### Evolutionary assembly of the neuronal program

**Figure 7:**
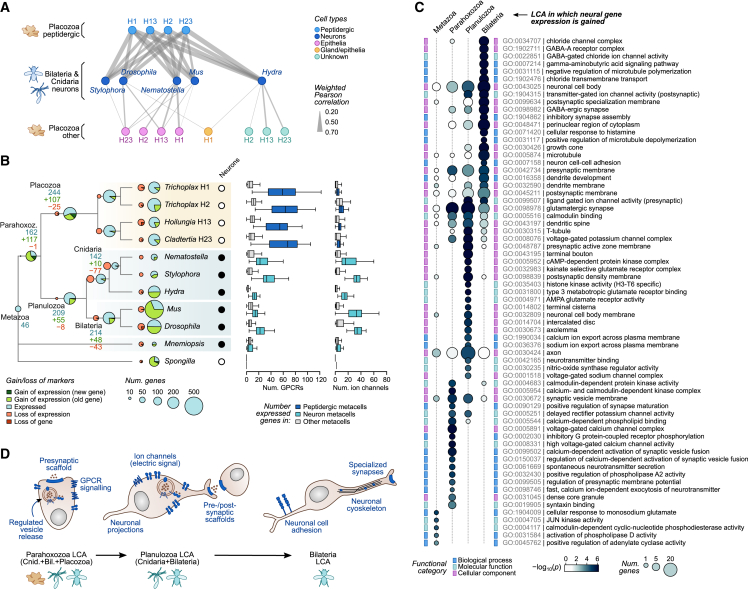
Stepwise evolutionary emergence of the neuronal gene expression program (A) Network summarizing pairwise similarities (weighted Pearson correlation) between neurons from cnidarians and bilaterians (middle) with placozoan cell types (top and bottom). Only similarities above 0.2 are shown. All pairwise cell type similarities across phyla are shown in Figure S3E. (B) Left, ancestral state reconstruction of neuronal gene expression programs across Metazoa. Pie charts indicate presence, gains and losses at each extant or ancestral node. Ancestral nodes are inferred using Dollo parsimony. Neuronal genes in each species are selected from single-cell atlases as having a FC ≥ 2 in at least 25% of the metacells annotated as neurons/neuron-like cells. Right, number of GPCRs and ion channels expressed in neuronal/neuronal-like metacells (threshold FC ≥ 2) versus non-neuronal metacells. (C) Gene ontology enrichments of gene gains in ancestral gene expression programs, based on annotations of the mouse orthologs. (D) Schematic representation of the major functional gains in the neuronal gene expression programs in early animal evolution. See also Figure S3.

We finally asked how conserved the expressed gene repertoires of placozoan cell types are compared with those of other animal phyla. To this end, we compared placozoan cell type transcriptomes with those of five other species from published whole-body cell atlases^15^^,^^66^^,^^67^^,^^68^^,^^69^^,^^70^^,^^71^^,^^72^ (Figure S3E). Using the same ICC-based strategy described above, we selected pairs of homologs with conserved expression across species. The comparison of cell type transcriptomes across species revealed global transcriptional similarities between placozoan peptidergic cells and cnidarian and bilaterian neurons (Figures 7A and S3E; Table S3).

To understand the evolutionary roots of neuronal gene expression programs, we reconstructed the patterns of gene expression gains and losses in neuronal/neuronal-like cells across early animal phylogeny (Figure 7B). Starting with ctenophore neurons^15^^,^^73^ and the recently proposed sponge neuroid cells,^68^ we identify only 46 genes with conserved expression between either of these lineages and Parahoxozoa (Placozoa, Cnidaria, and Bilateria). These genes are not particularly enriched in neuronal functions. In contrast, 162 genes are conserved in neuronal and peptidergic cells in the last common ancestor (LCA) of Parahoxozoa. These gained genes are involved in pre-synaptic membrane functions, including components of the synaptic vesicles and regulators of neurotransmitter release (Figure 7C). We reconstructed a neuronal gene expression program in the Planulozoa (Cnidaria + Bilateria) LCA involving 55 gene gains related to three key neuronal functions: post-synaptic scaffold components, neuronal cellular projections (axons, dendrites), and voltage-gated and ligand-gated ion channels (Figure 7C). Finally, the Bilateria LCA neuronal expression program gained 48 genes and was further enriched in specialized synaptic components and ion channels, particularly those related to GABAergic signaling, and microtubule cytoskeleton organization (Figure 7C). Overall, our results reconstruct the stepwise gain of neuronal functional modules in animal evolution, starting from a peptidergic secretory cell signaling system in the common ancestor of placozoans, cnidarians, and bilaterians (Figure 7D).

## Discussion

Our study presents a comprehensive panorama of the evolution of cell type programs across the placozoan lineage and in early animal evolution. The nine major placozoan somatic cell types are connected by intermediate transcriptional states. These states might represent transdifferentiating cell types, as observed in other early-branching metazoans like sponges.^32^ In addition, we identified groups of cells with transcriptional signatures of cell proliferation in each of these somatic cell types. Together, these observations suggest that placozoan cell type composition might be actively maintained by a combination of direct conversion and proliferation of differentiated cells. This is in sharp contrast with the fourteen peptidergic cell types. These cells not only do not show signatures of cell cycle and intermediate states with other somatic cells, but appear to derive from a distinct progenitor cell population with multiple molecular signatures typically associated with neurogenesis in cnidarians and bilaterians.^64^^,^^65^

Peptidergic cells share other similarities with cnidarian/bilaterian neurons. For example, they express large numbers of GPCRs (although very few ion channels) and unique combinations of post-translationally modified NPs, as well as an almost complete pre-synaptic gene module and NP-processing enzymes—albeit no post-synaptic module. Peptidergic cell types are defined by large TF modules, including a specific homeobox TF code reminiscent of that observed in nematode neuronal specification.^74^ Together, these observations suggest that complex neurosecretory signaling networks controlling collective cell behaviors are in place in placozoans, perhaps even akin to the peptidergic nerve nets observed in other animals.^75^ Systematic GPCR deorphanization^76^ will be necessary to define specific connections of this paracrine network. Related questions are which environmental cues trigger these networks (e.g., light,^77^ pH,^78^ population density, or glycine from food sources^79^) and also whether all or only some peptidergic cell types are sensory. The latter would involve some degree of receptor-effector cell specialization and two-cell neuronal-like circuits, a possibility that fits with our *in silico* peptidergic cell signaling network based on NP-GPCR structural predictions.

In terms of the evolutionary emergence of nervous systems, our findings indicate that key neuronal functional and ontogenetic gene modules originated in the context of non-neuronal secretory cell type networks, as proposed by the chemical brain hypothesis.^80^ These gene modules were combined with additional modules (the post-synaptic scaffold, genes involved in dendrite/axon formation, and ion channels generating fast electrical signals) in neuronal cells with synapses and projections. How ctenophore neurons fit in this scenario remains a major question as, despite having a largely peptidergic nervous system,^73^ they lack the conserved expression of the specific neuronal machinery and neurogenic program we report here for placozoans.

Finally, our study exemplifies how dense phylogenetic sampling of cell atlases will enable ever-more detailed reconstruction of ancestral cell states and cellular innovations.^81^ Beyond that, combined with *cis*-RE maps and genome sequence analysis, this multi-species atlas makes it possible to link regulatory sequence changes with the evolution of cell type transcriptional phenotypes.^82^ In the future, this systematic genotype-cellular phenotype mapping should help us better understand how cell type programs originate and evolve.

### Limitations of the study

Our study uncovers the diversity and evolution of placozoan cell types. There are several observations reported here that will deserve follow-up study to extend our understanding of placozoans cellular and molecular biology. Among these, we want to highlight: (1) the process by which transcriptional cell states intermediate to some of the terminal cell types are generated and whether this represents a case of *bona fide* transdifferentiation, (2) the role of specific NPs in placozoan collective cell behaviors and a systematic validation/discovery of their receptor targets via deorphanization experiments, (3) a further dissection of the molecular mechanisms underlying the specification of peptidergic precursors from lower epithelial cells and the differentiation of terminal peptidergic cell types, and (4) the spatial organization of the reported cell type diversity in placozoans. Finally, the phylogenetic position of placozoans among animals should be further explored in the future, as new genomes and phylogenetic methods become available.

## STAR★Methods

### Key resources table

**Table:** 

REAGENT or RESOURCE	SOURCE	IDENTIFIER
**Antibodies**		

anti-H3K4me2	Abcam	Cat#ab32356; RRID:AB_732924)
anti-H3K4me3	Millipore	Cat#07-473; RRID:AB_1977252

**Biological samples**		

*Trichoplax adhaerens* H1	Schierwater lab	N/A
*Trichoplax sp.* H2	Gruber-Vodicka lab	N/A
*Hoilungia honkongensis* H13	Schierwater lab	N/A
*Cladtertia collaboinventa* H23	Schierwater lab	N/A

**Chemicals, peptides, and recombinant proteins**		

TCO-PEG4-TFP Ester	Click Chemistry Tools	Cat#1398-2
Methyltetrazine-NHS Ester	Click Chemistry Tools	Cat#1128-25
Methyltetrazine-DBCO	Click Chemistry Tools	Cat#1022-10
DRAQ5	Thermo Scientific	Cat#62251
DAPT	Abcam	Cat#ab120633
LY411575	SelleckChem	Cat#S2714
Yhhu3792	Tocris	Cat#6599
Membrite-Fix 488/515	Biotum	Cat#30093-T
Proteinase-K	New England BioLabs	Cat#P8107S
EM-grade Paraformaldehyde	Electron Microscopy Sciences	Cat#15710
SPRI beads	Beckman Coulter	Cat#A63881

**Critical commercial assays**		

HCR v3 RNA FISH bundle for whole-mount	Molecular Instruments	N/A
HCR v3 RNA FISH bundle for cells in suspension	Molecular Instruments	N/A
Chromium Next GEM Single Cell 3' Kit v3.1	10x Genomics	Cat#1000269
Chromium Next GEM Chip G Single Cell Kit	10x Genomics	Cat#1000127
NEBNext Ultra II DNA Library Prep Kit	New England BioLabs	Cat#E7645L
UltraMicroSpin™ C18 columns	The Nest Group	Cat#SUM-SS18V

**Deposited data**		

Proteomics data for this project	PRIDE repository	PXD042821
Sequencing data for this project	GEO repository	GSE234601
Processed and reference data	Mendeley Data repository	https://doi.org/10.17632/bbpkbx968s.2
Code for this project	Github	https://github.com/xgrau/placozoa-cell-type-evolution-code

**Experimental models: Organisms/strains**		

*Trichoplax adhaerens* H1	Schierwater lab	N/A
*Trichoplax sp.* H2	Gruber-Vodicka lab	N/A
*Hoilungia hongkongensis* H13	Schierwater lab	N/A
*Cladtertia collaboinventa* H23	Schierwater lab	N/A

**Oligonucleotides**		

Clicktag barcoding primers for scRNA-seq samples multiplexing	Gehring et al.^83^	N/A

**Software and algorithms**		

IQ-TREE 2.1	Minh et al.^84^	http://www.iqtree.org/
MAFFT 7.475	Katoh and Standley^85^	https://mafft.cbrc.jp/alignment/server/
HMMER 3.3.2	Mistry et al.^86^	http://hmmer.org/
Metacell 0.37	Baran et al.^29^	https://rdrr.io/github/tanaylab/metacell/
STAR 2.7.9a	Dobin et al.^87^	https://github.com/alexdobin/STAR
MACS2 2.2.7.1	Zhang et al.^88^	https://github.com/macs3-project/MACS
CellRanger 6.1.1	v6.1.1 10X Genomics	https://support.10xgenomics.com/cloud-analysis/release-notes
BWA 0.7.17	Li and Durbin^89^	https://github.com/lh3/bwa
Possvm	Grau-Bové and Sebé-Pedrós^90^	https://github.com/xgrau/possvm-orthology/
deeptools 3.5.1	Ramírez et al.^91^	https://github.com/deeptools/deepTools
p4 1.5	Foster^92^	https://github.com/pgfoster/p4-phylogenetics
PhylobayesMPI 1.9	Lartillot et al.^26^	https://github.com/bayesiancook/pbmpi
ASTRAL 1.15.2.3	Zhang and Mirarab^93^	https://github.com/smirarab/ASTRAL
Seurat 4.1.1	Hao et al.^94^	https://satijalab.org/seurat/
clipkit 1.1.3	Steenwyk et al.^95^	https://github.com/JLSteenwyk/ClipKIT
MCL v4.137	Enright et al.^96^	https://micans.org/mcl/
MARE 1.0	Misof et al.^97^	https://bonn.leibniz-lib.de/en/research/research-centres-and-groups/mare
UFBS	Hoang et al.^98^	http://www.iqtree.org/
ModelFinder	Kalyaanamoorthy et al.^99^	http://www.iqtree.org/
diamond 2.0.14.152	Buchfink et al.^100^	https://github.com/bbuchfink/diamond
AUCell 1.16.0	van den Oord et al.^101^	https://github.com/aertslab/AUCell
TMHMM 2.0	Krogh et al.^102^	https://services.healthtech.dtu.dk/services/TMHMM-2.0/
SignalP 5.0b	Almagro Armenteros et al.^103^	https://services.healthtech.dtu.dk/services/SignalP-5.0/
SAMap 1.0.2	Tarashansky et al.^104^	https://github.com/atarashansky/SAMap
Iterative comparison of co-expression algorithm (ICC)	Tirosh and Barkai^31^	https://doi.org/10.1186/gb-2007-8-4-r50
WGCNA 1.71	Langfelder and Horvath^105^	https://horvath.genetics.ucla.edu/html/CoexpressionNetwork/Rpackages/WGCNA/
Broccoli 1.2	Derelle et al.^106^	https://github.com/rderelle/Broccoli
Proteome Discoverer 2.5	v2.5, Thermo Fisher Scientific	https://knowledge1.thermofisher.com/Software_and_Downloads/Chromatography_and_Mass_Spectrometry_Software/Proteome_Discoverer
Mascot 2.6	Perkins et al.^107^	https://www.matrixscience.com/mascot_support_v2_6.html
DeepNovo algorithm	Ntranos et al.^108^	https://github.com/nh2tran/DeepNovo

### Resource availability

#### Lead contact

Further information and requests for resources and reagents should be directed to and will be fulfilled by the lead contact, Arnau Sebé-Pedrós (arnau.sebe@crg.eu).

#### Materials availability

This study did not generate new unique reagents.

### Experimental model and study participant details

#### Animal culture

Specimens of *Trichoplax adhaerens* H1,^109^ *Hoilungia hongkongensis* H13^7^ and *Cladtertia collaboinventa* H23^12^ were obtained from stable cultures at the Schierwater laboratory. *Trichoplax* sp. H2^110^ specimens were obtained from stable cultures at the Gruber-Vodicka laboratory. The four placozoan species were maintained in 200 mm x 50 mm glass Petri dishes (Karl Hecht) with 35 ‰ artificial seawater (ASW; Red Sea Salt, Red Sea), which was changed (50% of the volume) every 15 days. Animals were fed *ad libidum*, on a weekly basis, with a suspension of the red alga *Pyrenomonas salina* (Wisłouch). Under this regime, animals divide asexually by fission. All individuals used for the different experiments were adult animals of diverse sizes. Algae were obtained from the University of Gottingen algae culture collection (SAG 28.87), and cultured in 250 ml flasks using Prov50 medium (NCMA). Animal and algal cultures were maintained at 23 °C with a 16:8 h light:dark regime.

#### Specimen dissociation and cell fixation

Around 200 adult animals were collected at a time and placed in a 2 ml Protein LoBind tube (Eppendorf), previously filled with 500 μl of ASW. Animals were pelleted by gentle centrifugation at 100 xg for 15 seconds in a tabletop centrifuge, and washed twice with 1 ml of calcium and magnesium-free artificial sea water (CMFSW: 10 mM Tris·HCl pH 8, 2.1427 mM NaHCO_3_, 10.7309 mM KCl, 426.0123 mM NaCl, 7.0403 mM Na_2_SO_4_). After washing, animals were suspended in 1 ml CMFSW plus 10 mM ethylenediaminetetraacetic acid (EDTA) and immediately placed on ice. Dissociation was performed by gently pipetting every 2 min for a total time of 10-12 min, until most of the sample was dissociated. After taking a 9 μl aliquot from the cell suspension to measure cell viability, the cell suspension was transferred to a 5 ml protein LoBind tube (Eppendorf), and immediately fixed. Cells’ viability was estimated by staining the cells with 1:10 of Acridine Orange/Propidium Iodide stain (Logos Biosystems #F23001), and observing using a Neubauer chamber on a Leica DMI6000 B fluorescence microscope with green and red filters. To fix the dissociated cells, we used a modified version of the ACME maceration protocol,^28^ that we called here ACMEsorb. Basically, cells were fixed by the addition of 3 ml of a fixative premix composed by 950 μl 1.2 M sorbitol, 300 μl glycerol, 300 μl glacial acetic acid and 450 μl methanol. Cells were fixed for 40 min at room temperature on a rotator set at 25 rpm. Fixed cells were collected by centrifugation at 2500 xg for 5 min at 4 °C using a swinging bucket rotor, suspended with 1 ml of freshly prepared ACMEsorb and filtered through a 40 μm strainer. An aliquot of the filtered cells was stained with 1:1000 4′,6-diamidino-2-phenylindole (DAPI) and counted on a Neubauer chamber. Sample volume was adjusted with ACMEsorb to get a concentration of 4 x10^6^ cells/ml and distributed into n aliquots of ∼400,000 cells in a volume of 100 μl.

### Method details

#### Single-cell RNA-seq methods

##### Sample Clicktag barcoding for scRNA-seq

Fixed cells were barcoded using a modified version of ClickTag.^83^ To optimize the labeling reaction in ACMEsorb fixative, we replaced the amine-reactive cross-linker TCO-NHS ([(4E)-cyclooct-4-en-1-yl] (2,5-dioxopyrrolidin-1-yl) carbonate) used in Gehring et al.^83^ by TCO-PEG4-TFP ((2,5-dioxopyrrolidin-1-yl) 3-[2-[2-[2-[2-[[(4Z)-cyclooct-4-en-1-yl]oxycarbonylamino]ethoxy]ethoxy]ethoxy]ethoxy]propanoate) (Click Chemistry Tools), which provides better stability towards hydrolysis in aqueous media. Barcoding DNA oligonucleotides (Clicktags) with a 5′-amino modifier (Integrated DNA Technologies) were activated by derivatization with methyltetrazine-NHS ester (MTZ-NHS) (Click Chemistry Tools), as originally described.^83^ For cell tagging, we used combinations of two different MTZ-derivatised oligonucleotides per sample. Fixed cells were pre-incubated for 5 min in the dark by addition of 4.5 μl of 1 mM TCO-PEG4-TFP. After pre-incubation, 12 μl of pre-mixed 100 mM Clicktags were added and mixed thoroughly by gentle vortexing. Cell labelling proceeded for 30 min at room temperature, protected from light, on a rotatory platform set at 25 rpm. Reactions were quenched by adding Tris·HCl pH 7 to a final concentration of 10 mM and methyltetrazine-DBCO (MTZ-DBCO) (Click Chemistry Tools) to 50 μM. After 5 min quenching, barcoded cells were pooled in a 2 ml protein LoBind tube (Eppendorf), according to the desired combination of Clicktags. Two volumes of Resuspension Buffer 1 (RB1; 1X PBS, 1% BSA, 0.8 M sorbitol, 80 U/ml Ribolock) were added to each pool of cells and gently mixed by inverting the tube three times. Cells were pelleted by centrifugation at 2000 xg for 5 min at 4 °C in a swinging bucket rotor, and washed once with 2 ml of RB1. After pelleting, cells were finally suspended in 900 μl RB1 plus 100 μl DMSO and kept at -80 °C until further processing.

##### Cell sorting and single-cell RNA-seq

Single-cell transcriptomes were obtained using the Chromium Single Cell 3′ Gene Expression kit v.3.1 (10x Genomics). For single-cell isolation, frozen samples were thawed on ice, and cells collected by centrifugation at 2000 xg for 5 min at 4 °C. Cells were washed once with 2 ml of Resuspension Buffer 2 (RB2; 1X PBS, 0.5% BSA, 80 U/ml Ribolock), pelleted again and finally suspended in 1 ml of RB2. Cell nuclei were stained with 1:300 DRAQ5 (Thermo #62251). Either 8,000 or 40,000 cells (for multiplexed experiments) were sorted into a well of a 96-well plate using a FACSAria II SORP cell sorter following the recommendations by 10x Genomics. Non-cellular particles were discriminated by selecting only DRAQ5-positive cells and doublets/multiplets were excluded using forward scatter width (FCS-W) versus forward scatter height (FCS-H). Cells were encapsulated immediately after sorting and barcoded cDNA and sequencing libraries were obtained following the recommendations by 10x Genomics. For Clicktags library preparation, Clicktags cDNA were separated from cellular cDNA after the cDNA amplification step, using differential size-selection purification with AMPure XP beads (Beckman Coulter). Clicktag sequencing libraries were prepared as previously described.^83^ Size distribution and concentration of the final libraries were calculated using TapeStation (Agilent) and Qubit (Invitrogen). Libraries were sequenced in an Illumina NextSeq 500 sequencer and high-output 75 cycles V2 kits (Illumina).

##### Notch signaling inhibition/activation experiments

About 500 animals (*Trichoplax* sp. H2) were incubated at room temperature and protected for light for 24 h in 10 cm plastic Petri dishes containing 20 ml of 35 ‰ ASW supplemented with either 50 μM DAPT, 10 μM LY411575 (both from 10 mM stock solutions in DMSO), 5 μM Yhhu3792 (from a 1 mM stock solution in DMSO), or 0.5% DMSO as the control condition. After incubation, animals were dissociated and used in a multiplexed scRNA-seq experiment using Clicktags as described above.

#### Hybridization Chain Reaction RNA FISH

##### Whole-mount Hybridization Chain Reaction RNA FISH and imaging

For whole-mount *in situ* HCR, animals were transferred to 22x22 mm glass coverslips with 150 μl of ASW and allowed to settle for at least 30 minutes. After removing most of the ASW, the coverslips were plunged into 4% paraformaldehyde in 1.5X PHEM buffer,^111^ at room temperature (RT) in a 3 cm plastic Petri dish. Fixation proceeded for 10 minutes at RT and overnight at 4 °C. Fixed animals were transferred to 1.5 ml protein LoBind tubes (Eppendorf), rinsed 3 times with ice-cold 1X PBS and dehydrated by rinsing 4 times during 10 minutes with ice-cold methanol and finally stored in methanol at -20 °C. Samples were rehydrated by successive transfers into 75% methanol – 25% PBS-T (1X PBS, 0.1% Tween-20), 50% methanol – 50% PBS-T, 25% methanol – 75% PBS-T and 100% PBS-T at RT. After rehydration, specimens were permeabilized with 0.01 mg/ml proteinase K (#P8107S, New England BioLabs), for 10 min at 37 °C. Hybridizations were performed with probes and reagents from Molecular Instruments (Los Ángeles, CA, USA) according to published protocols for whole-mount in situ HCR v.3.^112^ For membrane staining, animals were incubated in ASW with 1X MemBrite® Fix Pre-Staining Solution for 20 minutes at room temperature and then stained for 1 h with 1X Membrite Fix 488/515 (#30093-T, Biotum). After incubation, animals were rinsed twice with ASW and fixed as described above. Custom probes were designed for the following genes: TrH2_TrispH2_003088 (chymotrypsin), TrH2_TrispH2_010947 (fatty acid binding protein 4), TrH2_TrispH2_001718 (angiotensin I converting enzyme), TrH2_TrispH2_001704 (β-secretase 2), TrH2_TrispH2_007903 (calpain 9), TrH2_TrispH2_007301 (Klf13), TrH2_TrispH2_000422 (Delta receptor), HoiH23_PlH23_008135 (unknown protein), HoiH23_PlH23_003047 (Klf13). Samples were mounted either in glass bottom 8-well chamber slides (#80807, Ibidi) or SuperfrostPlus™ Gold Adhesion microscope slides (#K5800AMNZ72, Epredia), with mounting medium (#50001, Ibidi).

Images for calpain 9 localization were acquired in a Leica SP5 confocal microscope equipped with a 405 diode and DPSS 561 laser lines, using an HCX PL APO CS 20x 0.7 NA Dry objective. Scanner speed was set at 600Hz and bidirectional mode. 3D volume stacks were taken sequentially (in a frame-by-frame acquisition mode), with a 0.4 μm step size and 1024x1024 pixels image format, resulting in 505 nm pixel size. The rest of images were acquired in a Leica Stellaris 5 confocal microscope equipped with a 405 diode laser and a White Light laser, WLL (485–685nm) with a 80MHz repetition rate. A HC PL APO 20x 0.75 NA multi-immersion (glycerol) and a HC PLAN APO 63x 1.3 NA Gly CS2 objectives were used. Scanner speed was set to 600Hz, bidirectional. 3D volume stacks were taken sequentially (in a frame-by-frame acquisition mode) using 405nm (DAPI), 490nm (Alexa 488), 557nm (Alexa 546) and 633 nm (Alexa 647) excitation lines with three independent hybrid detectors (HyDs). Laser power and detector gains were set in each case, using control samples as reference. 3D volumes covering the whole specimens were taken with the 20x 0.75 NA objective, with a 1 to 2 micron Z -step size and 1024x1024 pixels image format. 3D volumes taken with the 63x 1.3 NA Gly objective were acquired with 0.3 micron Z -step and with 2048x2048 pixels image format, resulting in 90 nm pixel size. Images were analyzed with ImageJ v1.52 and Imaris Viewer x64 v9.9.1.

##### Hybridization Chain Reaction RNA FISH of cells in suspension and flow cytometry

For *in situ* HCR of cells in suspension, around 500 animals were collected and placed in a 2 ml protein LoBind Eppendorf tube with 500 μl of ASW. Animals were gently pelleted by centrifugation at 100xg during 15 seconds in a tabletop centrifuge and washed twice with 1 ml CMFSW. For dissociation, animals were suspended in 1 ml CMFSW-10 mM EDTA and placed on ice. Dissociation was performed by gentle pipetting during 10 min, after which cells were fixed by the addition of 1 ml of 2X fixative solution (8% paraformaldehyde in 3X PHEM buffer). Fixation proceeded for 2 hours at 4 °C. Fixed cells were collected by centrifugation at 180xg during 5 min at 4 °C in a swinging bucket rotor. Cells were washed four times with 2 ml PBS-T, centrifuging as before between washes. Cells were resuspended in 2 ml ice-cold 70% ethanol and permeabilized overnight at 4 °C. Cells were counted an aliquoted into five aliquots of ∼3x10^5^ cells. Hybridizations were performed with probes and reagents from Molecular Instruments (Los Ángeles, CA, USA) according to published protocols for in situ HCR v.3 for cells in suspension.^112^ Three samples were hybridized with a probe set against one of the following marker genes: TrH2_TrispH2_003088 (chymotrypsin), TrH2_TrispH2_010947 (fatty acid binding protein 4) or TrH2_TrispH2_001718 (angiotensin I converting enzyme). A fourth sample was hybridized in a multiplexed reaction with the three different probes sets, and the fifth one was subjected to the whole HCR procedure without any probe addition and was used as the unstained control. Flow cytometry sample acquisition was performed using a Cytek Aurora® 4 laser (V, B, YG, and R configuration) full spectrum cytometer (Cytek® Biosciences Inc., Fremont, California). Acquisition, spectral unmixing, and autofluorescence subtraction were performed using Cytek SpectroFlo® V3.1.0. software. Data analysis was performed using Cytek SpectroFlo® v3.1.0. software (Cytek® Biosciences Inc., Fremont, California) and FlowJo™ v10.9.0. software (BD Life Sciences).

#### Small peptide identification and analysis

##### Small peptide mass-spectrometry experiment

Protein extracts were prepared basically as described in Hayakawa et al.^38^ Samples were prepared by duplicate for each species, using about 1,000 animals per replica for *Trichoplax sp.* H2 and 500 animals for *H. honkongensis* H13 samples. Animals were collected in a 2 ml protein LoBind tube (Eppendorf) containing 500 μl ASW. Animals were pelleted by centrifugation at 100xg for 15 seconds at room temperature in a tabletop centrifuge. Immediately after removing supernatant, animals were mixed with 1 ml of acidified methanol solution (90% methanol, 9% ultrapure water, and 1% formic acid), together with ∼50 μl of glass beads (425-600 μm; #G8772, Sigma), and immediately subjected to 3 cycles of homogenization in a Mini-beadbeater 24 (dD Biolab) set at 3,000 rpm for 30 seconds at 4 °C with a 30 seconds pause between cycles. Homogenized samples were centrifuged at 13,000xg for 20 min at 4 to remove debris. Cleared supernatants were dried under vacuum and resuspended in 500 μl of 1% methanol with 0.5% formic acid. Solid phase extraction was performed with UltraMicroSpinTM C18 columns (3-30 μg capacity; The Nest Group), previously activated with 1x 400 μl methanol and 2x 300 μl 0.1% formic acid. Samples were eluted twice with 300 μl of 50% acetonitrile, 0.1% formic acid, vacuum dried and stored at -80 °C until their analysis.

Samples were analyzed using an Orbitrap Fusion Lumos mass spectrometer (Thermo Fisher Scientific, San Jose, USA) coupled to an EASY-nLC 1200 (Thermo Fisher Scientific, San Jose, USA) using a data dependent acquisition (DDA) method. Peptides were loaded directly onto the analytical column and were separated by reversed-phase chromatography using a 50-cm column with an inner diameter of 75 μm, packed with 2 μm C18 particles spectrometer (Thermo Scientific, San Jose, USA) with a 90 min chromatographic gradient. The mass spectrometer was operated in positive ionization mode in DDA, with acquisition in MS1 and MS2 level in Orbitrap mass analyzers. The method was driven by the “Top Speed” acquisition algorithm, which determined the number of selected precursor ions for fragmentation.

Digested bovine serum albumin (NEB, #P8108S) was analyzed between each sample to avoid sample carryover and to assure stability of the instrument. QCloud^113^ was used to control instrument longitudinal performance during the project.

#### Chromatin profiling experiments

##### ATAC-seq experiments

For the ATAC-seq experiments we basically followed the Omni-ATAC protocol.^114^ Animals were dissociated in CMFSW-10 mM EDTA as described in the previous section, cells were pelleted at 800 xg for 5 min at 4 °C and washed with 1 ml of CMFSW. All centrifugation steps were performed using a swinging bucket rotor. After resuspension in CMFSW, cells were filtered through a 40 μm strainer and counted on a Neubauer chamber using Acridine Orange/Propidium Iodide staining (Logos Biosystems #F23001) to estimate cell viability. Aliquots of different number of cells ranging from 50,000 to 500,000 were distributed into 1.5 ml protein LoBind tubes (Eppendorf) and pelleted at 800 xg for 5 min at 4 °C. After carefully removing supernatant, cells were suspended in 50 μl ice-cold lysis buffer and incubated on ice for 3 min. Nuclei were pelleted by centrifugation at 850 xg for 10 min at 4 °C, resuspended in 50 μl of tagmentation mix containing 2.5 μl of custom Tn5 transposase. Tagmentation proceeded for 35 min at 37 °C in a thermomixer with agitation at 1000 rpm. Tagmented DNA was purified with a DNA Clean & Concentrator-5 kit (Zymo Research). Libraries were pre-amplified by PCR using the NEBNext PCR mix (New England Biolabs) and quantified by qPCR. Size distribution and concentration of the final libraries were calculated using TapeStation (Agilent) and Qubit (Invitrogen). Sequencing was performed in an Illumina NextSeq 500 sequencer and mid-output 75 cycles V2 kits (Illumina).

##### ChIP-seq experiments

For each species, 200-350 individuals were crosslinked in 1% formaldehyde for 10 min at room temperature (RT) under vacuum. Crosslinking was quenched with 0.75 M Tris·HCl pH 7.5 for 5 min at RT under vacuum. Animals were washed with PBS, resuspended in 500 μL of Cell Lysis buffer (20 mM HEPES pH 7.5, 10 mM NaCl, 0.2% IGEPAL CA-630, 5 mM EDTA supplemented with a protease inhibitors cocktail), and incubated on ice for 10 min. Samples were centrifuged at max speed for 10 min at 4^o^C, and the resulting pellets were resuspended in Bead Beating buffer (20 mM HEPES pH 7.5, 10 mM NaCl, 5 mM EDTA supplemented with a protease inhibitors cocktail) and transferred to 0.2 mL tubes containing acid-washed glass beads (G8772, Sigma-Aldrich). Cells were lysed by vortexing 5 x 30 sec. The supernatants were transferred to a 1.5 ml sonication tube, SDS was added to 0.6% and samples were sonicated 3 cycles of 30 sec “on”, 30 sec “off” in a Bioruptor Pico (Diagenode, Seraing, Belgium) in order to generate 200-300 bp fragments. Chromatin was diluted with 5 volumes of Dilution buffer (20 mM HEPES pH 7.5, 140 mM NaCl), centrifuged at max for 10 min at 4^o^C, and stored at −80^o^C before use.

ChIP-seq was performed as previously described^15^ with small modifications. Briefly, an amount of chromatin equivalent to 100 ng of DNA per species was used per ChIP. Chromatin was incubated for 16 hours at 4 ^o^C with anti-H3K4me2 (#ab32356, Abcam) and anti-H3K4me3 (#07-473, Millipore) and recovered using a 1:1 mix of Protein A (16-661, Sigma-Aldrich) and Protein G magnetic beads (16-662, Sigma-Aldrich). Immuno-precipitated complexes were washed, reverse crosslinked for 3 hours at 68 ^o^C and treated with proteinase K. Immuno-precipitated DNA was eluted and purified using SPRI beads (A63881, Beckman Coulter). Libraries were prepared using the NEBNext Ultra II DNA Library Prep Kit (New England BioLabs) according to the manufacturer's protocol.

### Quantification and statistical analysis

#### Orthology inference

##### Genome-wide orthology assignment

For the four placozoan genomes, we used *Broccoli* 1.2^106^ to identify clusters of orthologous genes (step 3 in the *Broccoli* procedure) and pairs of orthologous genes (step 4), using the maximum-likelihood gene tree inference algorithm (based on *IQ-TREE*^84^) and a *k-*mer length of 10,000 to avoid the removal of paralogous sequences from the analysis. The input for this analysis were the protein sequences of the longest isoform per gene.

##### Gene family-specific phylogenetic analyses

We ran gene phylogenies to further refine the orthology assignments for selected gene families, including transcription factors. In this case, we collected a wider taxon sampling consisting of the translated peptide sequences from 37 metazoans (longest isoforms per gene; Table S4), which were queried using HMM profiles representing the DNA-binding regions of various transcription factor families (listed in Table S4) using hmmsearch from the *HMMER* 3.3.2 toolkit.^86^ For each gene family, the collection of homologous proteins was aligned in an all-to-all fashion using *diamond* v0.9.36^100^ (using the high sensitivity mode in blastp and up to 100 alignments per query) and divided into one or more low-granularity homology groups using the Markov Cluster Algorithm *MCL* v14.137^96^ (ABC mode using alignment bit-scores as weights, and a gene family-specific inflation parameter; see Table S4). Each of the resulting homology groups was then aligned using *mafft* 7.475^85^ (*E-INS-i* mode with up to 10,000 iterations), alignments were cleaned using *clipkit* v1.1.3^95^ (in *kpic-gappy* mode and a gap threshold = 0.7), and a gene phylogenetic tree was built using *IQ-TREE* v2.1.0.^84^ We ran each tree for up to 10,000 iterations until convergence threshold of 0.999 is met for 200 generations; the best-fitting substitution model was selected with *ModelFinder*^99^ among the commonly used LG, WAG and JTT models; statistical supports were obtained using the UFBoot procedure with 1,000 iterations.^98^ For each tree, the presence of outlier sequences was assessed using *treeshrink* v1.3.3^115^ (ran in a gene-wise manner and using the centroid rooting algorithm, and setting the a/b scaling factors to 10 and 1, respectively); if any outliers were identified, they were removed and the alignments and trees were recalculated. Orthology groups and ortholog pairs were then parsed from the final gene trees using *Possvm* 1.1,^90^ using the iterative gene tree rooting procedure for up to 10 steps. Human gene names were used to label the resulting orthogroups.

#### Phylogenomic analyses

##### Identification of single-copy orthologs to build a phylogenomic supermatrix

In order to build a phylogenomics dataset, we collected 81 animal and choanoflagellate genomes and transcriptomes (Table S1) and used *Broccoli*^106^ to obtain clusters of orthologous genes (henceforth, “orthogroups”) using the maximum-likelihood mode and keeping up to six homologs from each species in the gene tree reconstruction step. *Broccoli* identified 9,292 orthogroups, which were filtered to keep those which contained at least 75% of the species in the dataset and had a *Broccoli* clustering coefficient >0.9, resulting in a subset of 2,823 orthogroups. In parallel, we also repeated this procedure using only the 63 metazoans in the dataset, resulting in 29,665 and 5,453 orthogroups before and after filtering, respectively. At this step, orthogroups still retained internal paralogous sequences and were not amenable to build a phylogenomic matrix based on single-copy orthologs. In order to further refine the dataset, we built gene trees for each of these orthogroups (using the *mafft* and *IQ-TREE*-based procedure described in the previous section), and identified cases of internal paralogy using *Possvm* (rooting the trees with a two-step iterative rooting procedure). Each gene tree was then cleaned to obtain single-copy gene sets, using the following criteria: (i) we identified internal orthogroups within each gene tree using *Possvm* and, if we identified more than one orthogroup per tree, the largest one was retained; (ii) within the largest orthogroup, misplaced ‘outlier’ genes were identified, defined as genes with an infrequent nearest neighbor species in the rest of the single gene tree collection (the nearest neighbor species were defined using *k* = 1 closest neighbors in terms of phylogenetic distance, and outliers were determined with a frequency threshold < 1%); and (iii) within the largest orthogroup, recent duplications (e.g. species-specific) were collapsed and only the paralog with the longest protein sequence was retained (or, if tied, the one with the shortest phylogenetic distance to the root). This reduced the number of orthogroups to 2,794 single-copy gene sets for the Metazoa+Choanoflagellata dataset and 5,397 for the Metazoa-only dataset, which were then realigned using *mafft*, and a final set of gene trees produced with *IQ-TREE* (these individual trees were used for marker filtering, see below).

##### Concatenation, filtering and recoding of the supermatrix

These two sets of single-gene alignments (Metazoa-only and Metazoa+Choanoflagellata) were concatenated to produce two phylogenomic supermatrices. In the concatenation step, the list of orthogroups was filtered again to retain the most phylogenetically informative ones based on the following criteria: (i) they contained at least 3 genes from each of the main animal or outgroup clades of interest (Bilateria, Cnidaria, Placozoa, Porifera, Ctenophora, and, if relevant, Choanoflagellata); (ii) they contained at least 70% of the species in the dataset; (iii) in the corresponding gene-tree, the bilaterian, cnidarian and placozoan sequences formed a monophyletic clade (note that this criterion is agnostic to the internal topology of these clades); (iv) the alignment contained at least 50 aminoacid positions. This resulted in a 722 and 559 markers being selected for the Metazoa-only and Metazoa+Choanoflagellata supermatrices, respectively, containing 359,512 and 292,648 aminoacid positions each.

These matrices were further refined to retain markers with high phylogenetic information, using the *MARE* algorithm^97^ (weight parameter *α* = 2 and taxon weight = 100), resulting in 209 markers (93,453 positions) and 121 markers (57,774 positions) being retained for the Metazoa-only and Metazoa+Choanoflagellata supermatrices, respectively.

We also filtered the alignment supermatrices to retain compositionally homogeneous markers, as determined using the *compoTestUsingSimulations* function in the *p4* 1.4 toolkit.^92^ Specifically, we tested the compositional heterogeneity of each single-gene alignment using its respective gene tree as a reference (branch lengths were optimized in *p4* using the LG evolutionary model with four Γ rate categories, as this was the most common best-fitting model identified by *ModelFinder*), using 1,000 simulations to obtain a null distribution. Compositional homogeneity was determined for each marker using a *p* > 0.01 threshold. For the Metazoa-only and Metazoa+Choanoflagellata supermatrices, 140 markers (46,803 positions) and 62 markers (17,199 positions) were retained, respectively.

Each of the supermatrices described above was recoded using the Dayhoff6,^116^ SR4^117^ and SR6^117^ aminoacid recoding schemes. These were specified as follows. In Dayhoff6, 0 = AGPST, 1 = DENQ, 2 = HKR, 3 = ILMV, 4 = FWY, and 5 = C. In SR4, A = AGNPST, C = CHWY, G = DEKQR, and T = FILMV. In SR6, 0 = APST, 1 = DENG, 2 = QKR, 3 = MIVL, 4 = WC, and 5 = FYH.

##### Phylogenetic analyses of the supermatrix

We built species trees from each of the two versions the supermatrix (Metazoa-only and Metazoa+Choanoflagellata) with and without the compositional homogeneity filters (in the unfiltered set, we used the *MARE*-reduced version), as well as their unrecoded (aminoacid) and recoded versions (Dayhoff6, SR4 and SR6). This resulted in a total of 20 dataset combinations, to be analyzed with two phylogenetic inference methods: maximum-likelihood with *IQ-TREE* 2.1^84^ and Bayesian inference with *Phylobayes MPI* 1.9.^26^

For each dataset, we used the following models. For the unrecoded aminoacid supermatrices, we built trees using the LG substitution model^118^ with four Γ rate categories, empirical state frequencies observed from the data, and a CAT mixture model.^119^ In the Bayesian analyses, we used the full CAT model implemented in *PhylobayesMPI*.^26^ In the maximum-likelihood analyses, the C60 mixture model implemented in *IQ-TREE*^118^ was used instead. For the recoded matrices, an empirical GTR substitution matrix was used in combination with CAT profile mixture models (C60 in maximum-likelihood analyses). The DNA GTR model was used for the SR4 recoding (as each aminoacid group is mapped to the A, T, G and C nucleotides), and data-derived morphological GTR models were used for the other recoding schemes (which map aminoacid groups to states 0 through 5).

In all maximum-likelihood analyses, the *IQ-TREE* tree search step ran for up to 1,000 iterations until convergence threshold of 0.999 was met for at least 100 generations (which in all cases occurred within the first 200 iterations). Node supports were calculated using 1,000 UFboot iterations,^98^ which were individually recorded (-*wbt* flag in *IQ-TREE*).

In all Bayesian inference analyses, *PhylobayesMPI* was run for 3,000 generations in four parallel chains and using the CAT + GTR + Г4 model. *bpcomp* function was used to generate posterior consensus trees and check for chain convergence, removing the first 2,000 generations as burn-in. The final species tree results from the pair of chains with the lowest maximum bipartition discrepancy (*maxdiff*) and, in all cases, we requested maxdiff <0.1 to consider the chains have converged. If no pair of chains showed convergence after 3,000 generations, we ran additional 4 chains and extended all of them to 5,000 generations. In all cases, this resulted in convergence (maxdiff<0.1) for at least a pair of chains (in this cases, using 4,000 generations as burn-in).

To check the adequacy of the model to the different datasets, we used *PhylobayesMPI* to perform tests of compositional heterogeneity on all chains and using the same burn-in values (2,000/4,000 generations). We performed both posterior predictive tests of aminoacid diversity (*readpb_mpi -div*) and of compositional homogeneity across taxa (*readpb_mpi -comp*). For all tests, we use z-scores to summarize and report the outcome of the tests. These z-scores represent the deviation between the observed value and the mean of the null distribution and, therefore, a small absolute z-score indicates better adequacy of the model to the data. Using this criterion, we identified the metazoan-only (excluding choanoflagellates that show consistently high compositional heterogeneity) dataset without compositionally heterogeneous sites and with SR4/SR6 recordings as the ones with the best model fit.

In addition, we reanalyzed the *MARE*-filtered AA supermatrices using three possible partition schemes, using *IQ-TREE*: (i) gene-level partitions with partition-specific mixture models (optimizing the LG model + C20 in each partition, including +I, +F and +Г4 parameters if appropriate according to *ModelTest*); (ii) gene-level partitions without mixture models (only LG and +I, +F and +Г4 parameters if appropriate); and (iii) a heterotachy-aware edge-unlinked mixture model consisting of four site classes (i.e. the Heterogeneous evolution On a Single Topology model implemented in *IQ-TREE*).^120^

Finally, we selected six datasets for further analysis using fast-evolving site removal. Specifically, we selected the following supermatrices, for both the Metazoa and Metazoa+Choanoflagellata datasets: (i) AA *MARE*-filtered, (ii) AA compositionally homogeneous, and (iii) SR4-recoded *MARE-*filtered supermatrices. These datasets exhibited imperfect support for either of the the main phylogenetic hypotheses (Placozoa sister to Cnidaria and Placozoa sister to Cnidaria+Bilateria; Figure S1B), and we aimed to ascertain whether support for each topology would decrease after removing fast-evolving sites — a possible indication that the non-reduced dataset is affected by long-branch attraction. For example, the SR4 *MARE-*filtered Metazoan dataset exhibited had only 89% for the Placozoa sister to Cnidaria+Bilateria topology, but removal of fast-evolving sites increased this support to 100% (Figure S1H).

##### Phylogenetic analyses using supertrees

We used the collection of gene trees used to generate the Metazoa and Metazoa+Choanoflagellata supermatrices to create phylogenomic supertrees. First, we used the gene trees with one ortholog per species (prior to *MARE* filtering) to create a edge- and support-weighted supertrees with *ASTRAL* v1.15.2.3 in hybrid mode^93^ and assessed statistical supports for each branch with local posterior probabilities. Second, we used the paralogy-aware *ASTRAL-pro* algorithm^121^ to produce summary supertrees using the same collections of markers without removing intra-species duplicates (see above).

#### Single-cell RNA-seq data analysis

##### Transcriptome mapping and Clicktag deconvolution

We used *Cell Ranger* 6.1.1 (10X Genomics) to map reads and count unique molecular identifiers (UMIs) per gene per cell. We used the --*force-cells* flat to set a constant number of 20,000 per experiment (well in excess of the expected number cells for each case, as we intended to remove non-cells from the dataset using our own procedure; see below). We used whole gene bodies to guide the mapping of reads to the genome (*--transcriptome* flag). We also extended gene ranges to include proximal scRNA-seq peaks located downstream of each gene (in the region 5 kbp downstream of each gene, or less if another gene was closer to that peak). The extension of the 3′ regions of each gene was done to compensate for the low-quality annotation of UTRs in non-model species such as placozoans, as previously reported.^122^ To identify scRNA-seq peaks for the extension procedure, we mapped each scRNA-seq library to its respective genome with *STAR* 2.7.9a^87^ (tolerating up to 3 mismatching positions per read and 5 multi-mapping positions) and used *MACS2* 2.2.7.1^88^ to call peaks separately for reads mapping on each strand, with the *callpeak* utility and the following options: (i) an effective genome size equal to the ungapped genome length of each species (i.e. removing uncalled *N* bases), (ii) keeping up to 20 duplicates from different libraries (*--keep-dup 20*); (iii) retaining peaks with a false discovery rate threshold ≤ 0.01; (iv) retaining peaks of at least 30 bps (*--min-length 30*); (v) extending the read mapping region by 20 bp (*--extsize 20*); and (vi) disabling the modeling of peak extension for ChIP-seq libraries (*--nomodel* flag).

We discarded all cell barcodes with less than 100 UMIs/cell, upon examination of the distribution of UMIs per cell in each placozoan (Figure S2A). The scRNA-seq UMI matrices of all the *bona fide* cells was then converted to *MetaCell* 0.37 format^29^ for further processing in *R*.

Furthermore, our experiments included five Clicktag-multiplexed scRNA-seq libraries (namely: H1H13_1_ACME_CT_10x_10kc, H1H23_5_ACME_10x_10kc, H2H23_4_ACME_10x_10kc, Plac01_H1_H13_10XscRNAseq_10kc. and Plac02_H2_H23_10XscRNAseq_10kc), which included batches of cells from various pairs of species (e.g. *T. adhaerens* H1 and *H. hongkongensis* H13 for the first library). In these cases, we leveraged Clicktag counts to remove potential doublets, removing all cells that failed any of the following criteria, which were devised according to the methodology described by Chari et al.^123^ The results from these filtering steps are reported in Figures S3B and S3C.

First, we only kept cells with a Clicktag count >20 UMIs (to remove cells with not enough Clicktag counts to be reliably assigned to either species; these represented 6.5% of cells across all Clicktag datasets). Clicktag counts were determined as described by Chari et al.^123^: we mapped the reads to the Clicktag barcodes (8 bp barcodes + constant CAG sequences at the end) using *kallisto* 0.46.2,^124^ specifying the 10x v3 chemistry and tolerating one substitution per barcode; and used *bustools* 0.41.0^108^ to correct, sort and count the reads per cell, and obtain a final Clicktag UMI matrix.

Second, we compared the number of Clicktag counts for the most abundant barcode to the third most abundant barcode for each cell (given that we had used two barcodes per experimental batch, the abundance of the third most abundant barcode should be lower than the first and second ones, and correspond to a different batch of cells); and we kept cells where the first-to-third ratio of normalized Clicktag UMI counts was > 1.5. Likewise, we flagged as possible doublets all cells where the first and second most abundant Clicktag barcodes corresponded to different batches. Cells failing these criteria were flagged as potential doublets, and further classified as intra- or inter-species doublets depending on whether the two most abundant Clicktag barcodes corresponded to barcode pairs from the same/different species, respectively.

Third, we leveraged the fact that each of our Clicktag experiments corresponded to mixtures of different species to further remove cells that had similar scRNA-seq UMI counts in one species and the other. Specifically, we calculated the UMIs/cell against each of the two genomes, assigned each cell to the species where it had the highest UMIs/cell value, and discarded cells with similar UMIs/cell as potential doublets (threshold: relative UMI/cell ratio < 1.25 in either direction).

Fourth, we used *Seurat* 4.1.1^94^ to produce high-granularity Louvain clusters of cells based on their Clicktag count matrix, and removed all cells that fell within clusters that contained a high fraction (>70%) of cells flagged as potential doublets according to either of the previous criteria. These *Seurat* clusters were generated as follows: we used scaled and log-normalized counts with a scaling factor of 10,000 (*NormalizeData* function); selected variable features using default parameters (*FindVariableFeatures* function); ran a PCA and identified the 50 nearest neighbors of each cell (*RunPCA* and *FindNeighbors* functions with *k* = 50); and used this distance matrix to identify Louvain clusters (*FindClusters* function, with resolution = 1).

In summary, we only kept cells from Clicktag experiments that fulfilled these criteria: (i) had sufficient Clicktag counts to be confidently assigned to one experimental batch; (ii) had a strong enrichment of Clicktag counts from concordant pairs of barcodes, i.e. the most abundant barcodes corresponded to the same species and batch; (iii) had a strong enrichment in scRNA-seq counts for one of the pooled species over the other; and (iv) we used Louvain clustering to further identify cells which whose Clicktag count patterns were incompatible with these criteria.

##### Single cell transcriptome clustering

We used *Metacell* 0.37^29^ to select gene features and construct cell clusters (termed metacells). We selected feature genes using normalized size correlation^29^^,^^66^ threshold of -0.05 and normalized niche score^29^ threshold of 0.01, additionally filtering for genes with > 1 UMI in at least three cells and a total gene UMI count > 100 molecules (*mcell_gset_iter_multi* function in *Metacell*). For this step, we excluded (i) ribosomal proteins and histones based on their annotated Pfam domains, and (ii) batch-correlated genes, defined as genes for which their metacell-level expression fold change values were highly correlated with with the frequency of cells from certain experimental batches (samples) in that metacell (genes with Spearman correlation coefficient *ρ* > 0.5 were thus discarded). In total, we used 1,421 markers for *Trichoplax adhaerens* H1, 1,238 for *Trichoplax* sp. H2, 1,363 for *Holiungia honkongensis* H13 and 1,423 for *Cladtertia collaboinventa* H23. For *K*-nearest neighbours graph building we used *K* = 100 target number of edges per cell (*mcell_add_cgraph_from_mat_bknn* function), and for metacell construction we used K = 30, minimum module size of 10, and 1,000 iterations of bootstrapping with resampling 75% of the cells, and threshold α = 2 to filter edges by their co-clustering weight (*mcell_coclust_from_graph_resamp* and *mcell_mc_from_coclust_balanced* functions). This way we obtained an estimate of co-clustering frequency between all pairs of single cells and identified robust clusters of single or grouped metacells. For downstream analyses, we represent gene expression by computing a regularized geometric mean within each metacell (or cell type, where appropriate) and dividing this value by the median across metacells, as implemented in *Metacell*. We refer to these normalized gene expression values as fold change (FC) across the manuscript.

After defining the initial metacell set, we filtered out low-quality metacells using the following criteria: (i) the total number of UMIs per metacell was less than the *p* < 0.01 fraction of a standardized distribution of log_10_-transformed UMI counts; (ii) the median number of UMIs per cell in that metacell was less than the *p* < 0.01 fraction of a standardized distribution of log_10_-transformed UMI counts; (iii) the metacell had less than 10 genes with a fold change ≥ 1.5; (iv) the metacell did not have any transcription factor (defined based on Pfam domains) with a fold change ≥1.5; and (v) metacells in which too many cells originated from an overloaded Clicktag sample, which could result in intra-species doublets, were also removed (namely, metacells where the median first-to-third Clicktag count ratio was > 10, >50% of cells came from Clicktag samples, and >90% of cells came from the same Clicktag sample). This resulted in 12 metacells being removed in H1 (totaling 871 cells), 4 in H2 (265 cells), 5 in H13 (376 cells), and 9 in H23 (602 metacells). After excluding these cells, a new metacell clustering was calculated and expression FC values recalculated. Clusters of metacells, which we termed cell types (Table S2) were curated after inspection of the confusion matrix (calculated from the *K* = 100 nearest neighbors of each metacell) and its associated dendrogram (using a hierarchical agglomerative clustering approach^125^).

Two-dimensional projections of the metacells were produced using a force-directed layout based on the metacell co-clustering graph (*mcell_mc2d_force_knn* function in *Metacell*), using *K* = 20 nearest neighbors and a maximum confusion degree = 5.

Gene expression profiles across single-cells and metacells were visualized as heatmaps with the *ComplexHeatmap* 2.10.0^126^ *R* library. Unless otherwise stated, the cell type/metacell ordering was fixed according to the curated cell type tables described above; and gene order in the expression heatmaps was determined based on the highest fold change value per metacell. Genes were selected based on minimum differential expression per metacell/cell type, with a maximum number of markers per metacell/cell type selected in each case (the actual thresholds used in each heatmap are specified in the corresponding figure legend).

##### Characterization of transcriptomic signatures in intermediate cells

We validated our annotation of intermediate metacells with the *AUCell* 1.16.0^101^ *R* library, which was used to evaluate whether intermediate metacells exhibited higher-than-expected expression levels for gene markers specific to both of their (putatively) corresponding terminal cell types. First, we ranked each gene according to its fold change value in each metacell with the *AUCell_buildRankings* function. Second, we selected lists of cell type-specific genes for each pair of terminal cell types with a matching intermediate cell type, defined as having metacell expression FC ≥ 2.0 in that cell type (e.g., to evaluate the transcriptomic signal of intermediate lipophil-gland metacells, we selected genes specific to lipophil and gland terminal metacells). Third, we used the *AUCell_calcAUC* function to calculate the area under the curve (AUC) score of each metacell, for each of the terminal cell types. We expected intermediate metacells to exhibit relatively high AUC scores for both their terminal cell types.

We evaluated whether intermediate cells exhibited a conserved gene program comparable to that of other cell types. Specifically, for the intermediate cell types that could be identified across multiple species, we also evaluated whether their corresponding gene sets were conserved across species at a similar rate than those of their respective terminal cell types. To this end, for each intermediate cell type, we compared the fraction of intermediate markers shared across species (selecting orthology groups with a cell type-level FC ≥ 2.0 in each species) with the same fraction calculated from their corresponding terminal cell types, using a one-tailed exact binomial test. If intermediate cells shared a lesser number of markers across species, this could indicate that their transcriptional program could be more stochastic than that of terminal cell types.

Finally, we also evaluated the possibility that intermediate cells were doublets of two or more terminal cells. To that end, we tested whether the number of intermediate cells from each type was significantly different from the number of doublet cells one would obtain from random collisions (resulting from stochastic co-encapsulation) between terminal cell types, considering their relative abundance. We performed a two-tailed exact binomial test for each intermediate cell type, using the product of the fraction of both terminal cell types as the null probability. Significant deviations from this null probability in either direction would indicate that the number of cells from a particular intermediate cell type is not dependent on experimental conditions.

##### Characterisation of transcriptomic signatures in peptidergic cells

We counted the number of ion channels and GPCRs overexpressed in each metacell (FC ≥ 1.5). The ion channel category includes genes annotated with the following Pfam domains: ASC, Ion_trans, IRK, Lig_chan, Na_Ca_ex, Neur_chan_memb, P2X_receptor, PKD_channel, SK_channel, and VGCC_alpha2. The GPCR category includes the following Pfam domains: 7tm_1, 7tm_2, and 7tm_3.

The expression profile of the genes belonging to the pre-synaptic scaffold (pan-peptidergic, specific to certain peptidergic, other cell types, or not expressed, or not conserved) were manually recorded. The placozoan orthologs were obtained from the *Broccoli* database of orthologs used in the trans-phyletic analyses (see above), using well-characterised *M. musculus* and *D. melanogaster* sequences as queries. Specifically, the following genes were included: Synaphin/Complexin, Syntaxin Stx1a/1b/2/3/4, Snap25, Synaptotagmin Syt1/2/5/8, Synaptotagmin Syt7, Synaptotagmin Syt15, Ptpn5/Ptpn7/Ptprr, Ptprn/Ptprn2, Ptpdc1, Cacnb, Cacna2d, Cacn3, Cacna1A, Slc24a1, TrpA1, Kcnc, Kcnb/f/g/s, Kcnh1, Gucy2d, Munc13/Baiap3, Tomosyn, Rimbp, Rims, Cast/Erc, Spectrin, Caskin, Cask, Mint, Liprin, CamKII, Synaptophysin, Synaptogyrin-1, Synaptogyrin-2, Ncam, any homolog of the synaptic vesicle glycoprotein SV2, and any Cadherin domain-containing gene in placozoans (Table S4).

We also recorded the expression profile of neuropeptides homologs. First, we retrieved previously described sequences from *T. adhaerens*,^37^^,^^39^ and identified the relevant orthologs in other species based on the four-placozoan *Broccoli* orthology database (see above). Furthermore, we complemented this list with additional candidate genes, selected based on a sequence-based criteria, their neuropeptide-like expression pattern, and their conservation status in multiple placozoan species. Specifically, we selected groups of orthologs that fulfilled the following criteria: (i) homologs from at least two placozoans exhibited high expression specificity in peptidergic metacells (fold change ≥ 50 in at least one metacell); (ii) lack of transmembrane domains in all homologs, defined using TMHMM 2.0^102^; (iii) presence of a signal peptide (identifiable in at least one of the homologs), annotated with SignalP 5.0b^103^; (iv) none of the homologs contained any annotated Pfam domain; (v) we recorded the presence of neuropeptide-like signatures defined as follows: stretches of 3 to 10 residues flanked by one pair of lysines or arginines at the N-terminus and a pair composed of glycine and arginine or lysine at the C-terminus, as previously defined.^39^ We also assessed the homology of these candidates based on their similarity to previously published collections of invertebrate neuropeptides,^39^^,^^127^ assessed by local alignment with high-sensitivity *diamond blastp* searches. We used this homology search to add hypothetical names to the list of candidate neuropeptides, on a manual basis.

##### Characterization of cell cycle transcriptomic signatures

For each dataset, we identified individual cells with active cell cycle based on the expression of genes associated with the gene cycle modules (see below). Specifically, we retrieved all orthologous genes identified as part of the cell cycle modules in at least two species and calculated the fraction of UMIs per cell corresponding to this gene sets. We considered a cell to be in active cell cycle if it fell within the *p* ≥ 0.95 fraction of a standardized distribution of log_10_-transformed UMI counts, within each individual species.

##### Selection of gene pairs for cross-species transcriptome comparisons

We implemented the ‘iterative comparison of coexpression’ (ICC) algorithm in the following manner (Figures S3A and S3B). First, for a pair of species a and b, we retrieved gene expression matrices consisting of the metacell-level expression of matched one-to-one orthologs (defined using Broccoli, see above), representing using n genes across m_a_ and m_b_ conditions. Second, we calculated the Pearson correlation matrix of these two expression matrices, resulting in a n × n matrix where the diagonal represents a vector of correlation values between orthologous gene pairs. Third, the n-length correlation vector (range: −1 to +1) is used to obtain a vector of weights (range: 0 to +1; negative values are set to 0), which quantify the expression conservation of that pair of orthologous genes between species a and b, or EC_0_. Fourth, the EC_0_ vector is used to recalculate the n × n matrix using weighted Pearson correlation, a new weight vector EC_1_ is derived from its diagonal. This step is repeated for up to I iterations until the new weight vector equals the previous one (i.e. convergence is achieved), according to the following criterion: ∑(EC_i_ − EC_i-1_)^2^ < 0.05.

At this point, the EC_*i*_ vector represents the expression conservation values between each pair of one-to-one orthologous gene pairs. However, sets of genes with one-to-many or many-to-many paralogy relationships also may exist between species *a* and *b*. For each set of paralogous genes (e.g. gene *a*_*1*_ in species *a* with three paralogs *b*_*1*_, *b*_*2*_ and *b*_*3*_ in species *b*), we can select the most conserved paralogs (in terms of expression similarity) by selecting the gene pair with the highest *EC* value. To that end, the above procedure is repeated for each set of paralogous gene pairs between species *a* and *b*, adding all the possible paralog pairs at the end of the matched expression matrix (which implies including duplicated rows to accommodate all possible paralog pairs in that set, e.g. in the example above, *a*_*1*_ would need to be included three times in the matrix to match the three possible pairs with its three species *b* paralogs). In this context, orthologous gene pairs act as a reference set of co-expression values. For each set of paralogs, the pair with the highest EC_*i*_ value is then retained. In order to accelerate calculations, only 1,000 randomly selected pairs of orthologous genes are used in each of the paralog set-specific EC_*i*_ recalculations.

##### Cross-species cell type transcriptome comparisons

Pairwise comparisons between the gene expression profile of two species were performed using weighted Pearson correlation (as implemented in the *WGCNA* 1.71 *R* library^105^) of quantile-normalized gene normalized expression values (FC). Gene pairs were selected using the ICC procedure outlined above, including one-to-one orthologs and best paralog pairs; and the expression conservation values (EC score) between each pair were used as weights for the Pearson correlation. This procedure was applied to the following comparisons: cell type-level comparisons between the four placozoan species (Figure 3C), and broad cell type-level comparisons between the four placozoans and selected transcriptomes of poriferans (*Spongilla lacustris*), ctenophores (*Mnemiopsis leidyi*), cnidarians (*Nematostella vectensis*,^66^ *Stylophora pistillata*,^122^ and *Hydra vulgaris*^46^), and bilaterians (*Mus musculus*^70^^,^^72^ and *Drosophila melanogaster*^69^) (Figure S3). Single-cell clusterings and cell type annotations in cnidarians were obtained from Levy et al.^122^

In addition, we also used *SAMap* 1.0.2^104^ to calculate cell type mapping scores between all four placozoan species (Figure S3D). First, we created a database of pairwise protein alignments with *blastp* 2.5.0. Then, we used the cell-level UMI counts of each gene to calculate the *SAMap* mapping scores for each pair of cell types and metacells, using all cells within each cluster for score calculation.

Multi-species cell type clustering was performed using the UPGMA average algorithm implemented in *phangorn* 2.9.0,^128^ based on a Log-Det distance matrix obtained from the binarized gene expression of shared orthologs across cell types of different species (1 if gene is differentially expressed in that cell type with a minimum fold change; 0 if otherwise). Gene pairs were selected using the ICC procedure, using the shared pairs across all species in each comparison. Node supports were obtained from 1,000 iterations of Felsenstein’s bootstrap procedure^129^ as implemented in *phangorn*. This approach was followed to create cell type trees (using a fold change threshold ≥ 2.0 and retaining markers expressed in ≥ 70% of the cell types), at the level of broad (Figure 2E) and detailed cell types, with specific clustering of peptidergic cell types (Figure 4C). Cell type clusterings in cnidarians were obtained from Levy et al.^122^ Pairwise cell type similarities were visualized as heatmaps with the *ComplexHeatmap* 2.10.0^126^ *R* library or (ii) as weighted using the *igraph* R package,^130^ with nodes representing cell types and edge widths representing pairwise similarities, and using the Fruchterman and Reingold force-directed layout algorithm. Cell type clustering trees were visualized using the *ape* 5.6.2^131^ and *phangorn* 2.9.0^128^ *R* libraries.

##### Gene module analysis in placozoans

We used the metacell normalized gene expression fold change (FC) of each placozoan species to obtain gene modules using the *WGCNA*^105^ algorithm. First, we selected variable genes with a FC ≥ 1.25 in at least one metacell. Second, we calculated the gene co-expression matrix by calculating the Pearson correlation coefficient of each gene based on their metacell fold changes, and using the average hierarchical clustering algorithm and a soft power parameter = 7 (determined independently for each species using the *WGCNA pickSoftThreshold* function). Third, we used the hierarchical clustering dendrogram to define gene modules using the *cutreeHybrid* function in the *dynamicTreeCut* R library,^132^ maximizing granularity with a split parameter = 4 and ignoring clusters containing less than 10 genes; assigned each gene to one module with a correlation threshold ≥ 0.7; and we calculated the module Eigen vectors of each of the resulting modules in each metacell, using the *moduleEigengenes WGCNA* function. This resulted in 55 to 60 modules being identified in each species, containing between 6,341 and 8,474 genes (Figure S4).

Then, we grouped the modules across species based on the presence of shared orthologs to obtain multi-species module clusters, using a graph-based approach. Specifically, we calculated the Jaccard index between each pair of modules from different species using the list of orthogroups assigned to each module, and retained pairwise connections with a Jaccard index ≥ 0.1. Then, we identified cross-species module clusters using the *components* function in the *igraph* library, which was also used for visualization (Figure 4A). Finally, we refined the final module clusters, and manually annotated them according to their expression pattern across species as determined by inspection of the constituent single-species modules’ Eigen vectors across metacells (Figure S4) and the functional annotations of their constituent genes. This resulted in 34 cross-species module clusters, 30 of which represented by modules from three or more species. Out of these, 30 modules could be unambiguously assigned to specific cell types or functionally coherent groups of cell types (e.g. a module specific to *α*-peptidergic cells, or a general pan-peptidergic module); while the four remaining modules exhibited a cross-cell type expression profiles reflecting intermediate functions, such as cell cycle, ciliary genes, and meiosis genes.

The activity of each multi-species module cluster was scored across cell types by recording the fraction of genes belonging to each module that were differentially active in the cell types of individual species (defining active genes with a cell type-level expression FC ≥ 1.5).

##### Pan-metazoan neuronal gene module

We defined a pan-metazoan module of neuron/neuron-like associated genes, based on single-cell transcriptomic profiles from seven additional metazoan species, namely the cnidarians *Nematostella vectensis*,^66^ *Stylophora pistillata*,^122^ and *Hydra vulgaris*,^46^ the bilaterians *Mus musculus*^70^^,^^72^ and *Drosophila melanogaster*,^69^ the ctenophore *Mnemiopsis leidyi*,^15^ and the poriferan *Spongilla lacustris*.^68^ Specifically, we selected genes that were differentially expressed (expression FC ≥ 2.0) in at least 10% of the metacells annotated as neurons (or, in the case of *Spongilla lacustris*, the neuroids^68^). We also selected peptidergic cell-associated genes from each of the four placozoans using the same criterion. Then, we inferred the orthology relationship between these genes using *Broccoli* (same parameters as in the placozoan-specific analysis, see above; for this analysis, we also included *Amphimedon queenslandica* as a reference demosponge to compensate for the incompleteness of the *S. lacustris de novo* transcriptome), and mapped their genetic and transcriptomic evolutionary histories (ortholog gain/loss and expression gain/loss) on the metazoan tree of life using the Dollo parsimony procedure implemented in *Possvm*.^90^ According to this method, characters can be gained only once, and they are lost as many times as needed to explain their extant phylogenetic distribution. The number of gains/losses over species were visualized using the *ape* 5.6.2 *R* library.^131^

##### Comparison of peptidergic progenitors with neural developmental datasets

We characterized the transcriptomic similarities between placozoan peptidergic cells and developmental sc-RNAseq datasets from three query species: *M. musculus* (gastrula to pharyngula stage^44^), *N. vectensis* (gastrula to adult^45^^,^^66^), and *Hydra vulgaris* (regenerating adult^46^). For each of these species, we calculated normalized expression fold change values for each cell type and stage. Then, we grouped the cell types along neural developmental trajectories based on reported cross-stage similarities in the relevant studies. Specifically, we defined (i) five branching trajectories in mouse: neural crest (including the following cell types from the original annotation: Neural crest PNS glia, Neural crest PNS neurons, Olfactory sensory neurons, and Neural crest), forebrain/midbrain + developing neurons (Mesencephalon/MHB, Di/telencephalon, Retinal neurons, Retinal pigment cells, Retinal primordium, Forebrain/midbrain, Noradrenergic neurons, Motor neurons, Inhibitory interneurons, Di/mesencephalon inhibitory neurons, Spinal cord inhibitory neurons, Di/mesencephalon excitatory neurons, and Spinal cord excitatory neurons), neuron progenitors (Neuron progenitor cells and Intermediate progenitor cells), hindbrain + roof + anterior floor plates (Hindbrain, Roof plate, and Anterior floor plate) and spinal cord + posterior floor plate (Posterior floor plate, Spinal cord, Spinal cord dorsal and Spinal cord ventral); (ii) a four cell types continuously arising from neurosecretory precursors in *N. vectensis* (neurosecretory precursors, differentiated neuron cell types, cnidocytes, and gland cells); and (iii) two trajectories in *H. vulgaris* (neurosecretory precursors giving rise to neuron and gland cells, and nematocytes).

Then, we recorded whether selected markers of placozoan progenitor cells (Sox1/2/3, Sox4/11/12, Klf13, Insm1/2, Elav, Cpeb, Cecr2, Nanos1, and Hdac1) were overexpressed in any cell type from a given trajectory or stage in the query species (at expression FC ≥ 1.25). Furthermore, we recorded the number of TF and RNA-binding protein orthogroups shared between placozoan peptidergic progenitors from each species (at expression FC ≥ 1.5) and each query neural developmental trajectory as a whole (for branching trajectories, e.g. neural crest in mouse, we only considered the non-common part of the trajectory).

##### Functional enrichments in gene modules

We calculated functional enrichments of Gene Ontology (GO) terms using the *topGO* 1.0 *R* library.^133^ Specifically, we computed the functional enrichments based on the counts of genes belonging to each gene module relative to all annotated genes, using Fisher’s exact test and the *elim* algorithm for GO graph weighting. Functional enrichment tests of Pfam domain annotations were performed using hypergeometric tests as implemented in the R *stats* library, comparing the frequencies of presence of Pfam domains in each module to the same frequencies in the whole gene set (using unique domains per gene). For the multi-species placozoan modules, annotations for all four species were considered jointly. For the pan-metazoan neural module, annotations for the *Mus musculus* orthologs were used. Gene Ontology annotations were obtained from the November 2022 release of the Mouse Genome Database^134^ and mapped to the corresponding orthologs in other species using the *Broccoli* metazoan orthology set. Pfam annotations for each species were obtained with Pfamscan, using version 33.1 of the most regrettably decommissioned Pfam database.^135^

#### Neuropeptide data analysis

##### Mass-spectrometry data analysis

Data was analyzed using the Proteome Discoverer software suite (v 2.5, Thermo Fisher Scientific), and PEAKS software (v11, Bioinformatics solutions). For the Proteome Discoverer analysis, the Mascot^107^ search engine (v2.6, Matrix Science) was used for peptide identification. Data were searched against the predicted proteome of each species (*Trichoplax* sp. H2 and *H. hongkongensis* H13) plus the most common contaminants. The search was done considering amidated C-terminal, pyro-Glu for N-terminal glutamine or glutamic acid and oxidation of Methionine as variable modifications and the enzyme specificity was set as “None”. Precursor ion mass tolerance of 7 ppm at the MS1 and a fragment mass tolerance of 20 mmu at the MS2 level was used. False discovery rate (FDR) in peptide identification was set to a maximum of 1%. For the PEAKS analysis, the DeepNovo algorithm, that combines *de novo* sequencing and database search, was used. The database, the variable modifications, enzime specificity and FDR cutoff were the same as the ones used in the Proteome Discoverer analysis.

Then, we concatenated the lists of peptides detected using DeepNovo and database-based approaches, and ranked the peptides mapped to each candidate protein gene by their relative abundance in each search (percentiles of abundance as measured by the area under the peptide peak by each algorithm). For each peptide, we also recorded (i) the presence of pyro-Glu and amide post-translational modifications, (ii) its number of peptide spectra, and (iii) the number of matches in local alignments against their corresponding protein gene (with 1 mismatch). Finally, we recorded the following information for each of the protein genes of origin of the small peptides: (i) orthology group and conservation status across placozoans; (ii) expression level in the top three peptidergic cells in the relevant species (ranked by expression FC); (iii) presence of transmembrane domains using TMHMM 2.0^102^; (iv) presence of a signal peptide annotated with SignalP 5.0b^103^; (v) presence of neuropeptide-like signatures defined as follows: stretches of 3 to 10 residues flanked by one pair of lysines or arginines at the N-terminus and a pair composed of glycine and arginine or lysine at the C-terminus, as previously defined^39^; and (vi) possible homology to previously identified neuropeptides using local alignment with high-sensitivity *diamond blastp* searches against a database of previously described invertebrate neuropeptides.^39^^,^^127^ The complete list and sequences of the small peptides identified in each species (400 from 53 genes in H2, and 333 from 52 genes in H13), with all the above-mentioned metadata, is available in the Table S5.

##### Peptide-receptor complex modeling and docking analysis

With the aim of understanding the possible interactions between small peptides and their putative receptors (GPCRs and ASCs), we used a structural modeling approach to ascertain candidate docking pairs. To this end, we used *Trichoplax* sp. H2 as a model species. Given the extremely computational cost of these modeling analyses (it has 923 GPCRs, 11 ASCs and 400 small peptides, resulting in more than 300,000 possible combinations), we had to select a short list of candidate receptors and peptides for downstream analysis. The manual curation of the peptide list was based on the following criteria: (i) previously described placozoan neuropeptides^21^^,^^22^^,^^37^; (ii) for peptides detected in our proteomics experiment, their relative abundance; (iii) the presence amide or pyro-Glu of post-translational modifications in proteomics; (iv) high cell type-specificity of the protein gene of origin of the peptide (i.e. the propeptide); and (v) the conservation status of the peptide across species (prioritizing conserved peptides). In certain cases, we included peptides detected in *H. hongkongensis* H13 that could be aligned to H2 propeptide genes, with the assumption that they would be conserved (Table S5). In the case of GPCRs, we selected genes with (i) complete 7tm_1 or 7tm_2 Pfam domains (>90% length of the relevant HMM model); and (ii) high cell type-specificity, in order to prioritize GPCRs that would resolve cell type interactions (FC ≥ 5 in at least one peptidergic cell type, or 2 for non-peptidergic cell types). In the case of ASCs, we selected all genes with >90% complete HMM models (ASC Pfam domain). In total, we selected 60 small peptides and 207 candidate receptors (196 GPCRs and 11 ASCs), resulting in 12,420 possible combinations for further analysis.

Complexes between peptides and potential receptors (GPCRs and Amiloride Sensitive Channels) were modeled using a locally installed version of ColabFold - v1.5.2 (https://github.com/YoshitakaMo/localcolabfold), derived from the original ColabFold^41^ using the state of the art complex modeling tool Alphafold-multimer.^136^ The tool was used by following the joint GPCR-peptide modeling approach described by Lee et al.^43^ Each complex was generated by executing the "colabfold_batch" executable with the following ColabFold parameters: "templates," "amber," "num-recycle 20," "num-models 5," "model-type alphafold2_multimer_v3," "random-seed 16," and "use-gpu-relax". The model with the highest plDDT^42^ score was selected for each combination and then binding reliability was assessed using two different metrics: (i) the pDockQ function^137^ was used to distinguish acceptable from incorrect models; (ii) we used FoldX BuildModel^138^ to assess the quality of each binding interaction by mutating peptide residues to alanine *in silico* and evaluating the change in ΔG between the mutated and wild-type versions of the peptide. The rationale behind this analysis was that, in a realistic complex, mutating peptide residues to alanine should destabilize the binding and decrease ΔG values. The ΔΔG was measured using FoldX AnalyzeComplex (https://foldxsuite.crg.eu/). We selected receptor-peptide pairs for which models exhibited pDockQ scores higher than 0.23^137^ and FoldX ΔΔG values exceeding 0 kcal/mol (i.e. implying that the predicted docking is energetically favorable). The modeled complexes were visualized using ChimeraX 1.6.1.^139^

#### ATAC-seq and ChIP-seq data analysis

##### ATAC-seq and ChIP-seq data processing

The ATAC-seq libraries from each species were mapped to their respective genomes with *bwa* 0.7.17, using the *mem* algorithm.^89^ The resulting BAM files were filtered using the *alignmentSieve* utility from the *deeptools* 3.5.1 package,^91^ in order to filter out weak alignments (with minimum mapping quality or MAPQ = 30) and shifting the left and right ends of reads according to usual ATAC specifications (+4/−5 bp in the positive and negative strands, activated with the—*ATACshift* flag in *deeptools*). Duplicated reads were removed using *biobambam2* 2.0.87,^140^ and the resulting alignments were coordinate-sorted with the same tool. Then, we concatenated all the ATAC-seq libraries for each species.

The ChIP-seq libraries corresponding to H3K4me2 and H3K4me3 were mapped to their corresponding species with *bwa mem*, using the same procedure (except for the adjustment of read end coordinates specific to ATAC libraries).

Then we used *MACS2* 2.2.7.1^88^ to call ATAC peaks using the concatenated libraries for each species. Specifically, we used the *callpeak* utility to identify peaks from the filtered BAM files, with the following options: (i) an effective genome size equal to the ungapped genome length of each species (i.e. removing uncalled *N* bases), (ii) keeping duplicates from different libraries (*--keep-dup all* flag), (iii) retaining peaks with a false discovery rate ≤ 0.01, (iv) enabling multiple summit detection (*--call-summits* flag), and (v) disabling the modeling of peak extension for ChIP-seq libraries (*--nomodel* flag). The same procedure was also used to identify peaks of ChIP-seq H3K4me2 and H3K4me3 signal.

We evaluated the quality of our ATAC-seq and peak calling procedures by measuring the mapping rates of each species-specific library with the *flagstat* utility in the *samtools* 1.11 package,^141^ and the insert size distribution and fraction of reads in peaks using the *plotEnrichment* and *bamPEFragmentSize* utilities in *deeptools*. We also measured the strength of ATAC-seq, H3K4me2 and H3K4me3 signal around transcription start site regions (+/− 5 kbp, in 50 bp bins), using the *computeMatrix* and *plotProfile* utilities in *deeptools*.

##### Identification of regulatory elements and assignment to genes

For each species, we built collections of putative regulatory elements (REs) by combining the set of ATAC peaks (selecting *MACS2* peaks with *q-*value < 1×10^−6^ and homogenizing their length to 250 bp around the peak summit), and the promoter regions defined from the coordinates of each gene, defined as 200 bp upstream and 50 bp downstream of the transcription start site (TSS). This resulted in 19,286–25,164 REs per species. Then, we assigned each regulatory region to one or more genes based on their distance to TSS regions. Specifically, a given peak would be assigned to a particular gene if it lied within 2 kbp from its TSS. Peaks overlapping the promoter region of a particular gene were not assigned to any other gene. For this step, the TSS-defined promoter regions were supplemented with H3K4me3 ChIP-seq peaks assigned to their proximal genes. For the purpose of gene-regulatory element assignment, the peak sets were reduced to non-overlapping sets to avoid redundant regions. This resulted in 16,818-19,310 REs being assigned to 12,012-13,317 genes in each species (of which 9,908-11,119 were expressed). The above-mentioned genome coordinate operations were performed using the *GenomicRanges* 1.46 and *IRanges* 2.28 packages in *R*.^142^

Finally, we sorted the sets of REs according to the expression patterns of their associated genes in order to produce RE collections deemed to be active in specific cell types or gene modules. For the RE-cell type assignment step, we selected all regulatory elements associated with differentially overexpressed genes in each cell type, defined as having FC ≥ 2.0 in at least 25% of the metacells in that cell type. This procedure was performed three times: at the level of individual metacells, cell types, and broad cell types. For the RE-gene module assignments, we simply collected REs associated with genes belonging to each gene module.

##### Motif discovery and motif enrichment analysis

We used *HOMER* 4.11^143^ to identify novel motifs enriched in the regulatory of genes belonging to each multi-species gene module (calculated with *WGCNA*, see above). Specifically, we used the *de novo* motif finding tool *findMotifsGenome.pl*, with the following options: (i) the foreground consisted of the REs assigned to each cell type; (ii) the background consisted of REs that were not assigned to any of the metacells in that cell type; (iii) a constant peak size of 250 bp (equal to the RE size defined in the peak-gene assignment step, see above); (iv) attempting to identify motifs of lengths 8, 10 and 12 bp; (v) tolerating up to 2 mismatches in the global optimization step; (vi) and also trying to identify motifs from a known library with the *-mknown* flag. These sets of foreground and background sequences were built for each multi-species module, concatenating the regulatory regions of all four placozoans. The library of known motifs was compiled by concatenating the following publicly available resources: *HOMER* internal database,^143^ HOCOMOCO 10,^144^ The Human Transcription Factors database 1.01,^145^ and CisBP 2.0.^146^

The sets of *de novo* and known motifs identified by *HOMER* in each individual module were then concatenated into a single, multi-species motif library. This highly redundant motif collection was filtered and clustered as follows: (i) we removed motifs with *HOMER* enrichment *p*-values < 0.01; (ii) we only kept motifs with high contiguous information content (IC), defined as having IC ≥ 0.5 for at least four consecutive bases or IC ≥ 0.5 for two or more blocks of at least three bases; (iii) we only kept motifs that were identified by *HOMER* in at least five foreground sites; (iv) for each of the remaining motifs, we measured their pairwise sequence similarity by calculating the weighted Pearson correlation coefficient of the IC matrices of each motif, using the *merge_similar* function in the *universalmotif* 1.12.4^147^ *R* library with a similarity threshold = 0.80 for the hierarchical clustering and a minimum overlap of 6 bp between two motifs in the motif alignment step; and (v) a general motif archetype was defined from each cluster of similar motifs, based on the average of the position probability matrices of its aligned constituent motifs. This resulted in a single, multi-species collection of 2,009 motif archetypes. Each motif archetype was annotated with the names of its constituent motifs from known databases, which were also used to infer the putative structural class of its associated transcription factors.

Then, we aligned each motif archetype against the genomes of the four placozoan species using the *findMotifHits* function in the *monaLisa* 1.0 package.^148^ We retained motif alignments in regulatory elements that had an alignment score higher than the 98th percentile of its own background genome-wide distribution (obtained from scoring each motif against a random subset of 250 bp drawn from the whole genome, sub-sampled at 10%).

Finally, we calculated motif enrichment in the REs associated with specific gene modules. Specifically, we calculated the enrichment fold change from the frequency of occurrences of each motif in the set of foreground peaks for each gene module relative to its background, and assessed the significance of each positive enrichment using a hypergeometric test, correcting the resulting *p*-values with an empirical false discovery rate.

The motif enrichment profiles were visualized as heatmaps with the *ComplexHeatmap* 2.10.0^126^ *R* library. Motif order in these heatmaps was determined based on the highest enrichment value per gene module. Motifs were selected based on minimum enrichment per module (fold change ≥ 1.5, *p*-value < 0.1), with up to 20 motifs selected in each case.

All the necessary motif format conversions were performed using the *convert_type* function in *universalmotif*.^147^ Genomic sequence and coordinate manipulations in *R* were performed with the *Biostrings* 3.16^149^ and *GenomicRanges* libraries.^142^

#### Whole-genome alignments and regulatory sequence conservation analysis

We aligned the genome sequences of the four placozoan species using *Cactus* v2.1.1^150^ with default parameters, and converted the resulting multi-species alignment to four sets of alignments in MAF format with the *hal2maf* utility,^151^ each of them using each of the four placozoan genomes as the focal reference. Then, we processed these alignments to identify conserved regions using the *rphast* implementation of the *Phast* suite.^152^ Specifically, we used the *phyloFit* utility to initialize a null model of neutral change based on the four-fold degenerate codon positions of each genome’s coding regions, using a general reversible nucleotide transition matrix (REV) and a pre-defined species tree. Then, we used *phastCons* to optimize this model based on the expectation-maximization procedure, re-estimating the transition probabilities and tree parameters at each step. This model was generated based on the largest scaffold in the dataset (scaffold 1 from *T. adhaerens*, length = 13.2Mb), and then applied to all the smaller scaffolds in order to calculate (i) base-wise conservation/acceleration *p*-values based on the neutral model described above using the likelihood ratio test implemented in *phyloP*^33^; (ii) and, again with *phyloP*, conservation/acceleration *p*-values calculated for each regulatory element identified in each species (including TSS-based promoter regions; see “*ATAC-seq and ChIP-seq data analysis*” section).

Finally, we used the whole-genome alignment information to characterize the conservation status of the non-coding regulatory regions across and within species. Specifically, the regulatory regions from a given species were considered to be conserved in another one if (i) their coordinates could be lifted across genomes using the *halLiftover* tool^151^ (peaks closer than 10bp were considered jointly for the purpose of coordinate lifting); (ii) they fell within a well-aligned region of >20 bp in the reference and query genomes. Pairs of regions regions fulfilling these criteria were recorded as edges in an homology graph, and grouped into clusters of homologous regulatory elements using the *components* utility in *igraph*. The presence/absence status of each cluster of homologous regulatory elements in the four extant species was then used to reconstruct their gain/loss pattern over the four-species tree using the Dollo parsimony procedure as implemented in *Possvm*. According to this method, characters can be gained only once, and they are lost as many times as needed to explain their extant phylogenetic distribution. We have applied this procedure to the following datasets: (i) presence/absence of groups of orthologous genes in each genome; (ii) presence/absence of homologous regulatory elements across genomes; (iii) activity of orthologous genes in conserved cell types; (iv) activity of homologous regulatory regions across cell types; (v) presence of transcription factor binding motifs in genes expressed across cell types. The number of gains/losses over species were visualized using the *ape* 5.6.2 *R* library.^131^

Within each species, the each regulatory region was further classified as accelerated, neutral or slow-evolving based on their regional *phyloP* scores^33^ and *p*-values (slow-evolving: score > 0 and *p* < 0.001; acceleration: score < 0 and *p* < 0.001; neutral otherwise).

##### Additional resources

The dataset can be interactively explored (and data downloaded) in https://sebelab.crg.eu/placozoa_cell_atlas/. We show there detailed 2D projections and gene expression maps for all four placozoan species. Additional interactive functionalities include inspecting the expression of individual genes or groups of genes, and retrieving specifically expressed genes in metacells or cell types, using user-defined thresholds. Finally, pairwise species cell types similarities can be explored using different distance metrics and varying sets of orthologs, and it is also possible to inspect genes supporting each cell type similarity.

## Data Availability

• Raw DNA and RNA sequencing data is available in GEO repository under accession number GEO: GSE234601. Mass spectrometry proteomics data is available in ProteomeXchange Consortium via PRIDE: PXD042821. Processed data and annotation tables can be downloaded in Mendeley Data: 10.17632/bbpkbx968s.2 and interactively explored in https://sebelab.crg.eu/placozoa_cell_atlas/. • All code to generate the single cell atlases, perform cross-species comparisons and analyze gene modules is available in GitHub: https://github.com/xgrau/placozoa-cell-type-evolution-code. Unless otherwise specified, scripts are based on R version 4.1.2 and Python version 3.9.12. • Any additional information required to reanalyze the data reported in this paper is available from the lead contact upon request.

## Supplemental information

Table S1. Phylogenomic datasets, related to Figure 1 (A) Taxon sampling and data sources of the 81 genomes and transcriptomes used in the phylogenomic analyses. (B) List of selected marker genes in the Metazoa + Choanoflagellata dataset, recording the number of genes and the species represented in that orthologous group; and whether the marker has been included in the supermatrix alignments based on a number of criteria: sufficient species coverage, sufficient length, sufficient taxon-specific species coverage, and whether the gene is included in the *MARE*-reduced or compositionally homogeneous datasets. (C) Likewise, for the Metazoa-only dataset. (D) Genes IDs included in each of the orthologous groups that are used in the Metazoa+Choanoflagellata dataset. (E) Likewise, for the Metazoa-only dataset.

Table S2. Cell type classification, related to Figure 2 (A–D) Cell type annotations of the numbered metacells for *Trichoplax adhaerens* H1, *Trichoplax* sp. H2, *Hoilungia hongkongensis* H13, and *Cladtertia collaboinventa* H23, respectively. Cell type annotations are provided at two levels: broad cell types (lipophil, fiber, gland, epithelia/gland, epithelia, peptidergic, intermediate, meiotic and unknown) and more fine-grained cell types (e.g. including each peptidergic subtype).

Table S3. Gene modules, related to Figures 3 and 5 (A) List of gene modules identified in each placozoan species, with their corresponding multi-species module, and a curated annotation based on its functional enrichments and the broad cell types where each module is most active. (B) Gene module membership of each placozoan gene, including the membership coefficient score from *WGCNA* and gene annotations. (C) Functional enrichments of gene sets belonging to each of the multi-species gene modules, based on Pfam and Gene Ontology (GO) annotations. Enrichment *p*-values for Pfam domains were obtained from a hypergeometric test. Enrichment *p*-values for GO terms were obtained from Fisher’s exact tests. Annotations from all four placozoan species have been used for these enrichments. (D) List of markers shared between placozoan peptidergic progenitors (defined as being expressed in the placozoan ancestor of this cell type, based on Dollo parsimony and FC ≥ 1.5 in extant species) and the neural developmental trajectories of three other species (mouse, *N. vectensis*, and *H. vulgaris*), as shown in Figure 6H. Developmental trajectories for each species are denoted by acronyms listed at the top of the table. For each set of markers (listed at the metazoan orthogroup level), we specify the annotation of its placozoan orthologs, and whether the marker is a TF, an RBP, or a chromatin regulator (see STAR Methods). (E) Functional enrichments of gene sets from the pan-neural gene module with gained expression in key nodes of the metazoan tree: Metazoa, Parahoxozoa (Cnidaria+Bilateria+Placozoa), Planulozoa (Cnidaria+Bilateria), Placozoa, and Bilateria. Annotations from the *Mus musculus* orthologs have been used for these enrichments.

Table S4. Comparative genomic analyses, related to Figures 3, 5, and 7 (A) Taxon sampling and data sources of the 37 metazoan genomes and transcriptomes used in the comparative genomic analyses. (B) List of Pfam domains associated with transcription factor families, which were used to retrieve metazoan homologs of each family and to perform dedicated phylogenetic analyses. Gene-family-specific *MCL* inflation and HMM scoring parameters are provided. Lists of Pfam domains corresponding to RNA-binding proteins, chromatin regulators, and ion channels are also included. (C) Orthology groups produced for the four placozoan species, including putative gene names derived from homology to human genes or dedicated transcription factor annotations, if available. (D) Likewise, for the metazoan species used in the reconstruction of the pan-neural gene module (the four placozoans, *Nematostella vectensis*, *Stylophora pistillata*, *Hydra vulgaris*, *Mus musculus*, *Drosophila melanogaster*, *Mnemiopsis leidyi*, *Spongilla lacustris*, and *Amphimedon queenslandica*). (E) Orthology pairs for the four-species placozoan orthology dataset. (F) Phylogeny-informed classification of placozoan transcription factors. (G) List of neural-like genes identified in placozoans (using *Trichoplax* sp. H2 as a reference), associated to three categories: the pre-synaptic scaffold, the post-synaptic scaffold, and various genes associated with neural progenitors in other species. Orthologs were obtained from the metazoan-wide set of orthologs, using known *M. musculus* and *D. melanogaster* genes as queries. (H) Phylogeny-informed classification of placozoan GPCRs, naming orthology groups with human gene names if available, and including the peptide sequence.

Table S5. Neuropeptides identified by mass-spectrometry, related to Figure 5 (A) Complete list of small peptides identified by proteomics in *Trichoplax* sp. H2 and H. *hongkongensis* H13. For each peptide, we indicate the species from which it was identified; its source (database search in ProteomeDiscoverer: DB Search PD; *de novo* DeepNovo: DeepNovo; database search DeepNovo:DB Search; homology DeepNovo: homolog; previously known peptide: NA); its source gene according to each search algorithm; known annotations from that gene, including if it matches a previously known placozoan propeptide or has homology to other invertebrate neuropeptides (labelled “hypothetical”); the orthogroup of the source gene and its conservation across placozoans; its sequence and length; its relative abundance (percentile abundance within each search; see STAR Methods); the number of PSMs detected; the number of alignments to the source gene and its orthologs in other placozoans (with up to one mismatch); its post-translational modifications (PTMs); the cell type where the source gene has a highest expression, including the corresponding FC and UMI fraction per 10^3^ values; and a factor indicating whether the source orthogroup has the following propeptide signatures: presence signal peptide (“wSP” if present, “nSP” if absent), lack of transmembrane domains (“nTM” or “wTM”), and presence of stretches of 3 to 10 residues flanked by one pair of lysines or arginines at the N-terminus and a pair composed of glycine and arginine or lysine at the C-terminus (“signature” or “no-signature”). In the case of peptides identified by DeepNovo, we also indicate the MS correlation and global confidence values. (B) Curated list of 60 neuropeptides selected for downstream analysis. For each peptide, the same fields as in Table S5A are included. (C) Curated list of GPCR and ASC receptors selected for downstream analysis. For each receptor, we indicate its class, orthology to human genes if available, whether it has been selected as a peptidergic-specific gene or specific to other somatic cell types, and its expression FC in the top three cell types where it is expressed. (D) List of predicted dockings between peptides and receptors, the peptide and receptor names and annotations (same as above), the pLDDT AlphaFold2 score of model quality, pDockQ docking scores and its associated positive predictive value, and the FoldX ΔΔG value of the positive interaction. (E) Same as (D), but including all tested pairs.
